# Cloning-free CRISPR/Cas system facilitates functional cassette knock-in in mice

**DOI:** 10.1186/s13059-015-0653-x

**Published:** 2015-04-29

**Authors:** Tomomi Aida, Keiho Chiyo, Takako Usami, Harumi Ishikubo, Risa Imahashi, Yusaku Wada, Kenji F Tanaka, Tetsushi Sakuma, Takashi Yamamoto, Kohichi Tanaka

**Affiliations:** Laboratory of Molecular Neuroscience, Medical Research Institute (MRI), Tokyo Medical and Dental University (TMDU), Tokyo, 113-8510 Japan; Laboratory of Recombinant Animals, MRI, TMDU, Tokyo, 101-0062 Japan; The Center for Brain Integration Research (CBIR), TMDU, Tokyo, 113-8510 Japan; Department of Mathematical and Life Sciences, Graduate School of Science, Hiroshima University, Hiroshima, 739-8526 Japan; Department of Neuropsychiatry, Keio University School of Medicine, Tokyo, 160-8582 Japan; FASMAC Co. Ltd, Kanagawa, 243-0021 Japan; JST, CREST, Saitama, 332-0012 Japan

## Abstract

Although the CRISPR/Cas system has enabled one-step generation of knockout mice, low success rates of cassette knock-in limit its application range. Here we show that cloning-free, direct nuclear delivery of Cas9 protein complex with chemically synthesized dual RNAs enables highly efficient target digestion, leading to generation of knock-in mice carrying a functional cassette with up to 50% efficiency, compared with just 10% by a commonly used method consisting of Cas9 mRNA and single guide RNA. Our cloning-free CRISPR/Cas system facilitates rapid one-step generation of cassette knock-in mice, accelerating functional genomic research by providing various *in vivo* genetic tools.

**Electronic supplementary material:**

The online version of this article (doi:10.1186/s13059-015-0653-x) contains supplementary material, which is available to authorized users.

## Background

Although gene-targeted knockout and knock-in mice are invaluable tools for understanding the functions of genes *in vivo*, the production of such genetically modified mice has relied on gene targeting in embryonic stem cells, which is a complicated and time-consuming process [1]. The recent development of the clustered regularly interspaced short palindromic repeat (CRISPR)/CRISPR-associated protein (Cas) system, a genome editing technology, has allowed for the direct manipulation of the genome in mouse zygotes *in vivo* (*in vivo* genome editing) with extremely high efficiency, enabling the highly convenient and ultra-rapid one-step generation of genetically modified mice without embryonic stem cells [2,3].

A flood of studies using CRISPR/Cas-mediated *in vivo* genome editing have reported the production of knockout mice [4-6] and knock-in mice carrying single nucleotide substitutions combined with oligo DNA donors [5,7,8]. In contrast, there has been only one report on the successful production of knock-in mice carrying reporter gene cassettes [9], essential tools for analyzing complex tissues such as brain *in vivo* [10], and the efficacy of the targeted insertion of the reporter gene was only about 10% [2,3,9,11]. The low success rates of gene cassette knock-in limit the applicability of CRISPR/Cas-mediated *in vivo* genome editing.

The CRISPR/Cas system was initially reported as an adaptive immune system in bacteria, consisting of three components including Cas9 nuclease and two small RNAs, CRISPR RNA (crRNA), which guides the Cas9 complex to the target sequence, and trans-activating crRNA (tracrRNA), which binds to crRNA and forms a ribonucleoprotein complex with Cas9 nuclease [12]. When it was harnessed as a genome editing tool [13,14], the dual-crRNA:tracrRNA was engineered as a chimeric single guide RNA (sgRNA) [13]. The CRISPR/Cas system consisting of two components - Cas9 nuclease and sgRNA - is the most common approach in the field of genome editing due to its enhanced convenience and robust targeting [15]. However, it is still unknown whether the commonly used sgRNA works more efficiently than the dual-crRNA:tracrRNA, especially for the production of knock-in mice carrying reporter gene cassettes.

Here, we show the highly efficient generation of knock-in mice carrying a functional gene cassette by a cloning-free CRISPR/Cas system using Cas9 protein combined with chemically synthesized dual-crRNA:tracrRNA.

## Results

### Generation of highly active guide sequence

**Figure 1:**
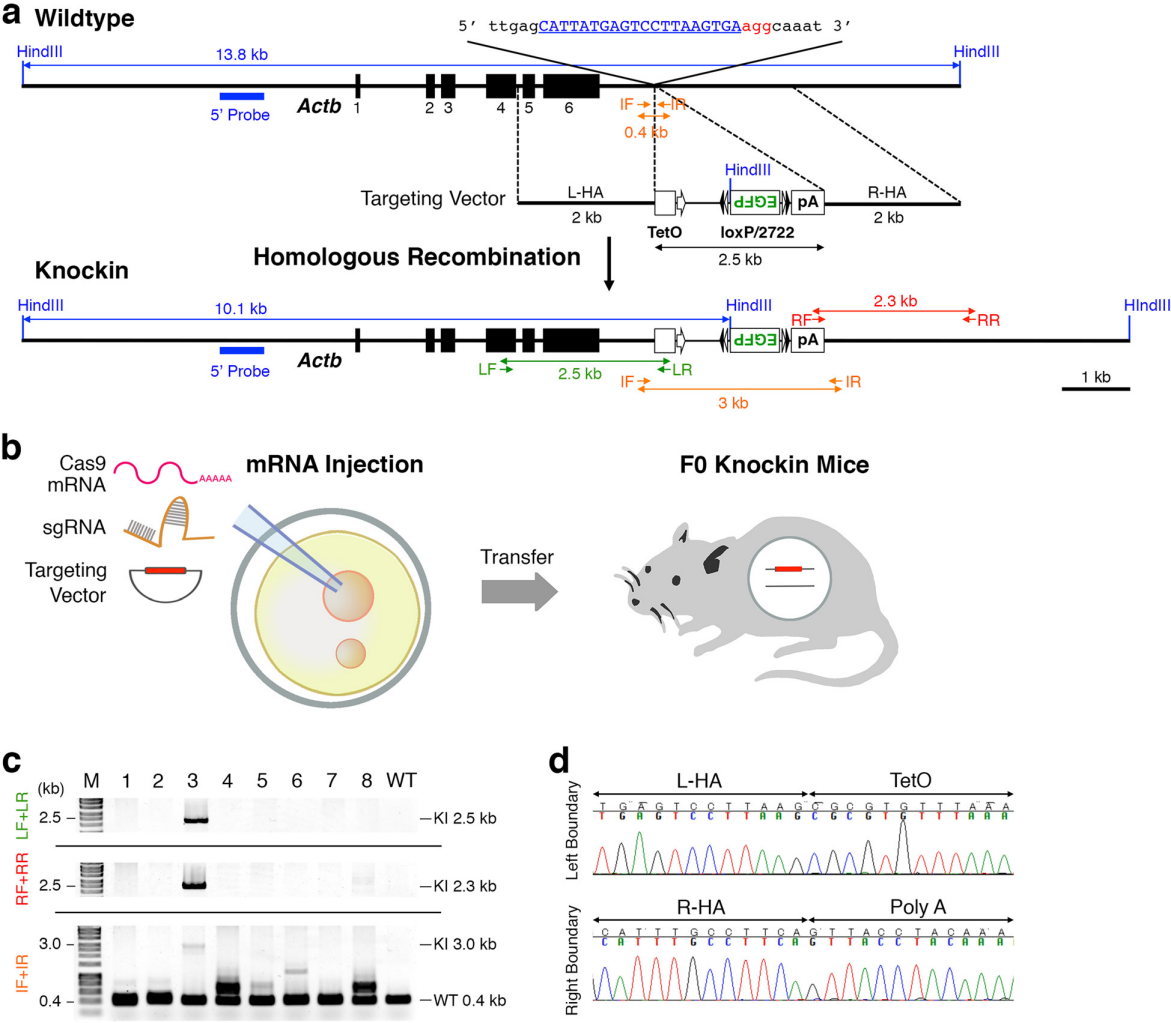
Generation of knock-in mice carrying gene cassette by sgRNA combined with Cas9 mRNA injection. **(a)** Targeting strategy for the generation of *Actb*-TetO-FLEX-EGFP-polyA knock-in mice. **(b)** Schematic diagram of pronuclear injection of Cas9 mRNA, *Actb* sgRNA and TetO-FLEX-EGFP targeting vector. **(c)** PCR screening of knock-in newborns derived from pronuclear RNA injection. **(d)** Sequences of boundaries between *Actb* and TetO-FLEX-EGFP-polyA cassette. IF, internal forward primer; IR, internal reverse primer; KI, knock-in; LF, left forward primer; LR, left reverse primer; RF, right forward primer; RR, right reverse primer; L-HA, left homology arm; R-HA, right homology arm; M, molecular marker; WT, wild type.

In a previous study, we demonstrated that the insertion of a transgene downstream of the *Actb* polyadenylation signal allowed for sufficiently high levels of gene induction [16,17]. Thus, we chose the *Actb* locus as a model to be targeted for the generation of knock-in mice carrying a functional gene cassette. We first designed the guide sequence targeted to the locus 800 bp downstream of the mouse *Actb* polyA signal (Figure 1a) and inserted it into a bi-cistronic expression vector pX330 plasmid [18,19] containing sequences encoding Cas9 and sgRNA backbone sequences. Then, we determined its high activity of DNA digestion *in vitro* using a single-strand annealing (SSA) assay with episomal plasmid vectors containing a split luciferase gene and *Actb* target sequences in human HEK293T cell lines (Figure S1 in Additional file 1), and a Cel-I assay in mouse Neuro2A cell lines to target the endogenous mouse chromosome (Figure S2 in Additional file 1).

To further test the activity of *Actb* sgRNA *in vivo*, we injected Cas9 mRNA with *Actb* sgRNA - both *in vitro* transcribed with T7 RNA polymerase using PCR templates amplified from an *Actb* pX330 plasmid [11] - into one-cell stage mouse zygotes. We obtained 12 newborn mice and found that almost all the newborns were bi-allelically targeted mutants (Figure S3 and Table S1 in Additional file 1). Thus, the *Actb* sgRNA we designed is highly active both *in vitro* and *in vivo* for target digestion and subsequent induction of non-homologous end-joining (NHEJ).

### Generation of reporter knock-in mice by sgRNA combined with Cas9 mRNA

As a model for knock-in mice carrying a functional gene cassette, we designed reporter mice highly expressing enhanced green fluorescent protein (EGFP) from the endogenous *Actb* locus only in a specific cell population intersectionally defined [20] by the expression of Cre recombinase and tetracycline transactivator (tTA). We constructed a 10.5 kb targeting vector for the mouse *Actb* locus containing a 2.5 kb TetO-FLEX-EGFP-polyA cassette (Tet operator (tetO) sequence concatemers fused to a minimal cytomegalovirus (CMV) promoter, beta-globin intron, inverted EGFP flanked by two pairs of loxP and lox2722 (FLEX switch) and polyA), and 2.0 kb left and right homology arms of the *Actb* locus (*Actb*-TetO-FLEX-EGFP-polyA; Figure 1a).

Next, we injected the circular *Actb*-TetO-FLEX-EGFP-polyA targeting vector together with *Actb* sgRNA and Cas9 mRNA, both *in vitro* transcribed as above, into one-cell-stage mouse zygotes (Figure 1b). We injected the mixture (5 ng/μl Cas9 mRNA, 2.5 ng/μl *Actb* sgRNA, and 10 ng/μl of the targeting vector) into pronuclei as previously described [9]. We obtained eight newborn mice and screened them by PCR with three different primer pairs (Figure 1a) and tail genomic DNA to detect homologous recombination (HR). We found one correctly targeted knock-in mouse carrying a TetO-FLEX-EGFP-polyA cassette at the *Actb* locus (Figure 1c), whereas the endogenous *Actb* loci were still targeted by NHEJ in all the newborn mice (Table 1; and Figure S4 in Additional file 1). The efficiency of the targeted insertion of the transgene by pronuclear injection was 12.5%, consistent with the previous report (Table 1) [9]. Next, we sequenced cloned PCR products of the left and right boundaries between *Actb* and the TetO-FLEX-EGFP-polyA cassette and found the precise knock-in of the cassette, as we designed (Figure 1d). These results confirm that the knock-in mouse carrying a functional gene cassette can be generated by CRISPR/Cas-mediated *in vivo* genome editing, although its efficiency is low even when using highly active sgRNA for NHEJ.

**Table 1:** **Generation of reporter knock-in mice by Cas9 mRNA or protein combined with sgRNA or crRNA/tracrRNA**

**Cas9**	**Guide RNA**	**Injected**	**Transferred (%)**	**Newborn (%)**	**Targeted (%)**	**Knock-in (%)**
mRNA	sgRNA	86	58 (67.4)	8 (13.8)	8 (100)	1 (12.5)
Protein	sgRNA	135	112 (83.0)	19 (17.0)	5 (26.3)	0 (0)
Protein	crRNA and tracrRNA	107	65 (60.7)	11 (16.9)	9 (81.8)	5 (45.5)

Percentages were calculated using the number in each column as the numerator and the number in the column to its left as the denominator except for Knock-in. Percentages of Knock-in were calculated using the number in the Knock-in column as the numerator and the number in the Newborn column as the denominator. Note that the results of protein injections with high concentrations of guide RNAs are shown. All results are shown in Tables S2 and S3 in Additional file 1.

### Generation of reporter knock-in mice by sgRNA combined with Cas9 protein

To digest target genomic DNA, Cas9 mRNA injected into pronuclei of mouse zygotes must be exported to the cytoplasm, translated into protein, and imported into the nucleus again. This long process [21] might delay target digestion, leading to a low rate of HR. Consistent with this idea, a recent *in vitro* cellular study reported that the target genomic DNA was almost immediately digested by the direct delivery of a Cas9 protein-sgRNA complex compared with the delivery of plasmid DNA expressing Cas9 and sgRNA [22]. Further, an *in vivo* study in mice and zebrafish revealed that the direct delivery of a Cas9 protein-sgRNA complex into embryos led to highly efficient generation of knockout animals [23]. Thus, we hypothesized that direct delivery of a Cas9 protein-RNA complex into the pronuclei of mouse zygotes and subsequent rapid digestion of target genomic DNA might improve the efficiency of knock-in mouse generation.

**Figure 2:**
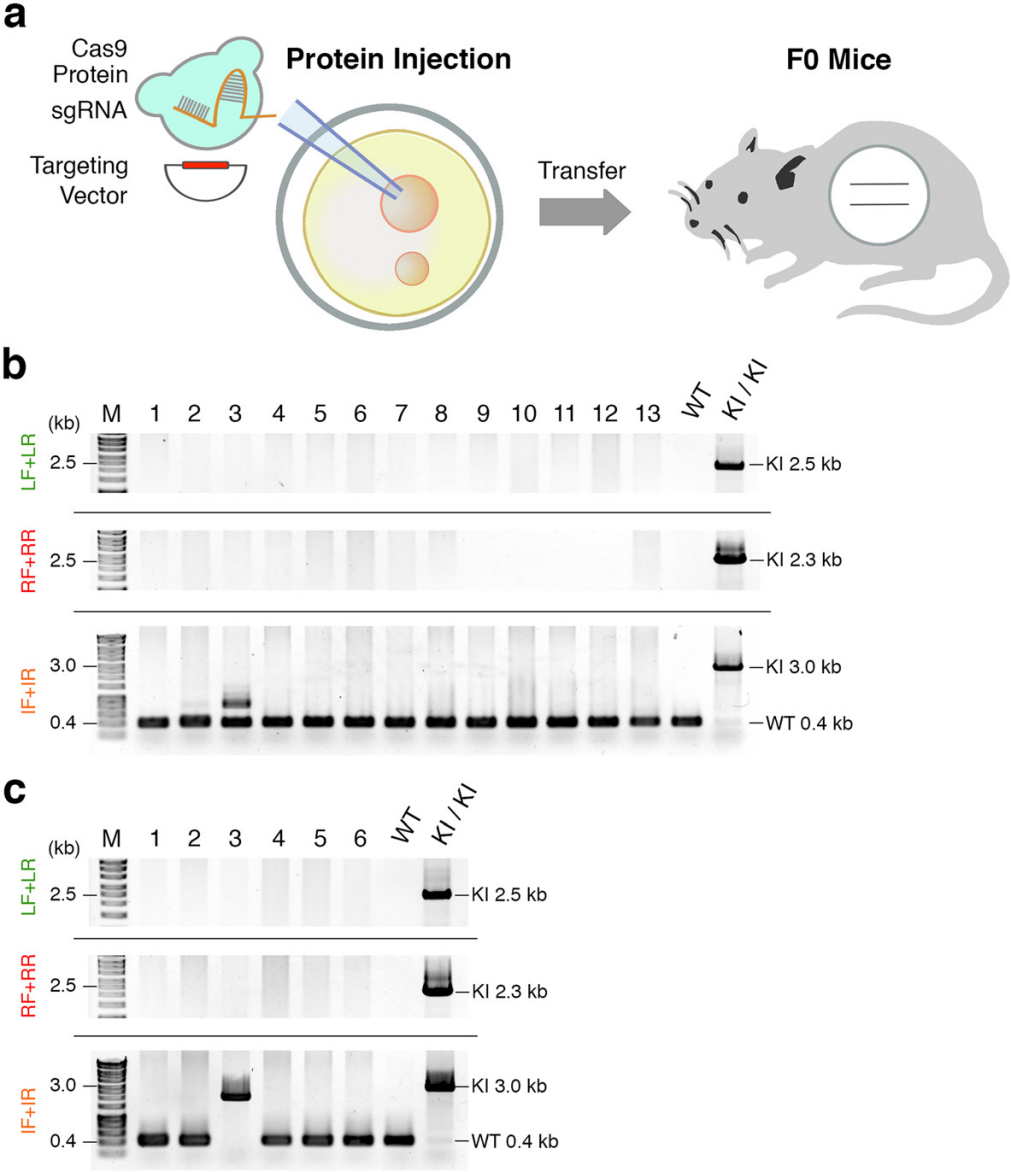
Generation of knock-in mice carrying a gene cassette by sgRNA combined with Cas9 protein injection. **(a)** Schematic diagram of pronuclear injection of Cas9 protein, sgRNA, and TetO-FLEX-EGFP-polyA targeting vector. **(b,c)** PCR screening of knock-in newborns derived from 30 **(b)** or 100 ng/μl **(c)** Cas9 protein injection. Note that the size of the IF+IR PCR product in lane 3 in **(c)** is lower than that of the knock-in PCR product. IF, internal forward primer; IR, internal reverse primer; KI, knock-in; LF, left forward primer; LR, left reverse primer; RF, right forward primer; RR, right reverse primer; M, molecular marker; WT, wild type.

First, we injected a mixture consisting of 30 or 100 ng/μl Cas9 protein, 2.5 ng/μl *Actb* sgRNA, and 10 ng/μl targeting vector into the pronuclei of zygotes (Figure 2a). We obtained 39 newborn mice, although none carried the TetO-FLEX-EGFP-polyA cassette at the *Actb* locus (Figure S5 and Table S2 in Additional file 1). We also found that the endogenous *Actb* locus was targeted by NHEJ in only one mouse (Figure S6 and Table S2 in Additional file 1). Next, we injected a mixture containing a higher dose of sgRNA (30 or 100 ng/μl Cas9 protein, 25 ng/μl *Actb* sgRNA, and 10 ng/μl targeting vector) into the pronulei of zygotes. We obtained 19 newborn mice, but again none carried the TetO-FLEX-EGFP-polyA cassette at the *Actb* locus (Figure 2b,c; Table S2 in Additional file 1). We also found the endogenous *Actb* loci were targeted by NHEJ in 5 out of the 19 mice (Figure S7 and Table S2 in Additional file 1). Collectively, although we generated 58 newborn mice by direct pronuclear delivery of a Cas9 protein-sgRNA complex, we unexpectedly did not obtain any knock-in mice carrying a functional gene cassette (Table 1).

### Cloning-free CRISPR/Cas system

**Figure 3:**
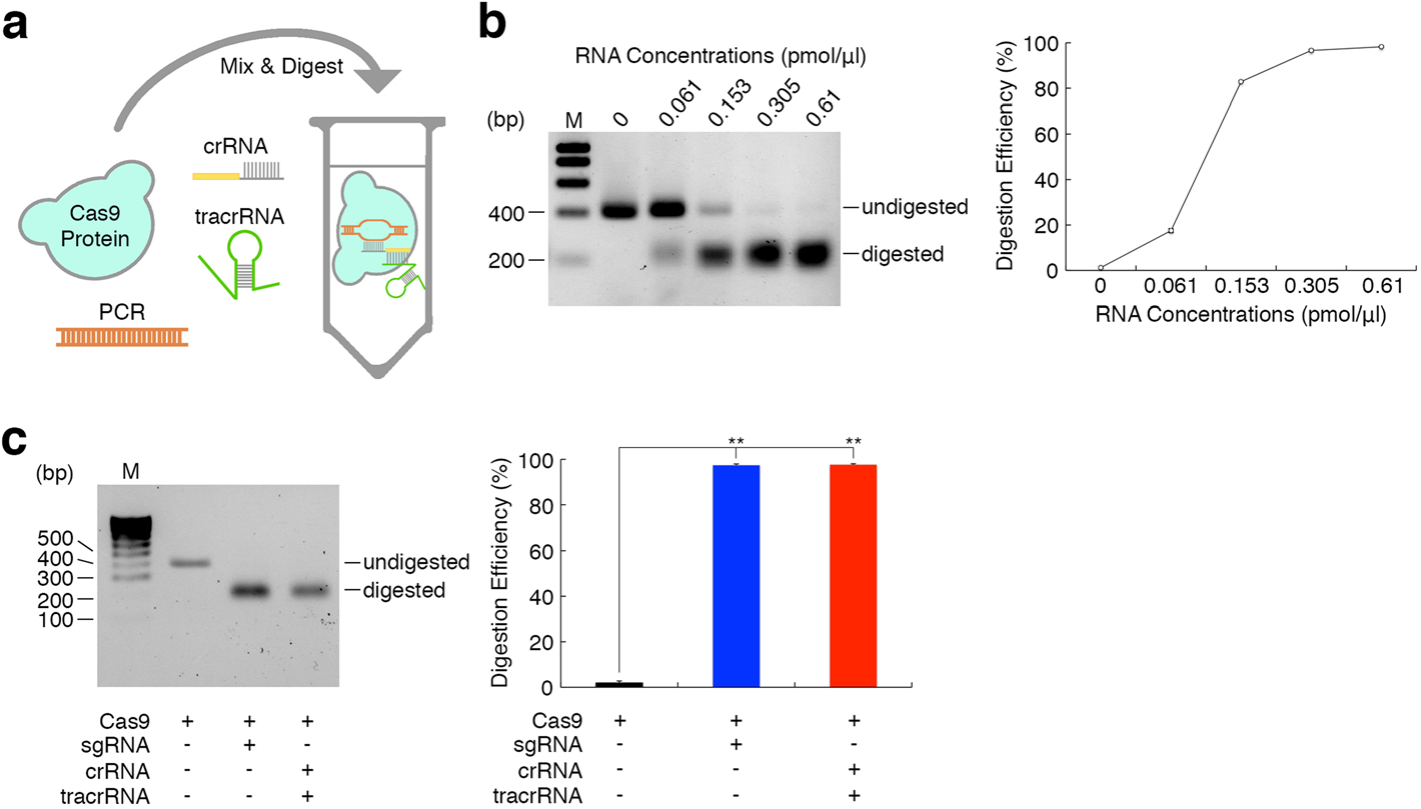
Cloning-free CRISPR/Cas system. **(a)** Schematic diagram of the *in vitro* digestion assay with Cas9 protein. The PCR product is the *Actb* PCR product amplified from wild-type mouse genomic DNA with internal forward and internal reverse primers. **(b)** The dose-dependency of target digestion by chemically synthesized crRNA/tracrRNA with Cas9 protein (n = 3). RNA concentrations represent the concentration of each crRNA and tracrRNA. **(c)** The efficiencies of target digestion by Cas9 protein only, Cas9 protein and sgRNA, and Cas9 protein and chemically synthesized crRNA/tracrRNA (n = 4, respectively). Statistical significance was determined by one-way ANOVA and *post hoc* Tukey-Kramer test. **P < 0.01. M, molecular marker.

To explore highly efficient knock-in systems based on the Cas9 protein, we tested the initial form of the CRISPR/Cas system consisting of three components including Cas9, crRNA and tracrRNA (dual RNAs). The short lengths of crRNA and tracrRNA are appropriate for their chemical synthesis, enabling a cloning-free CRISPR/Cas system when combined with Cas9 protein. Taking advantage of the cloning-free CRISPR/Cas system, we tried to simplify the evaluation process of CRISPR activity generally performed in cultured cells. We developed a cell-free *in vitro* digestion assay (IDA) system [13] using the target *Actb* PCR product, Cas9 protein, and chemically synthesized dual RNAs (Figure 3a). We chemically synthesized *Actb* crRNA containing identical 20 nucleotide guide sequences of *Actb* sgRNA and tracrRNA. We tested the target digestion activities of various concentrations of chemically synthesized dual RNAs combined with Cas9 protein by IDA. We found that chemically synthesized dual RNAs efficiently digested target DNA in a dose-dependent manner with Cas9 protein (Figure 3b). We then compared the target digestion activities of chemically synthesized dual RNAs with *in vitro* transcribed *Actb* sgRNA. We found that both the sgRNA and dual RNAs combined with Cas9 protein digested the target *Actb* PCR product with efficiencies of over 95% (Figure 3c). These results suggest that the digestion activity of CRISPR/Cas can be quickly and conveniently evaluated using IDA without cultured cells [13], and chemically synthesized RNAs can be used instead of *in vitro* transcribed sgRNA, enabling a cloning-free CRISPR/Cas system.

### Highly efficient generation of reporter knock-in mice by dual-crRNA:tracrRNA combined with Cas9 protein

**Figure 4:**
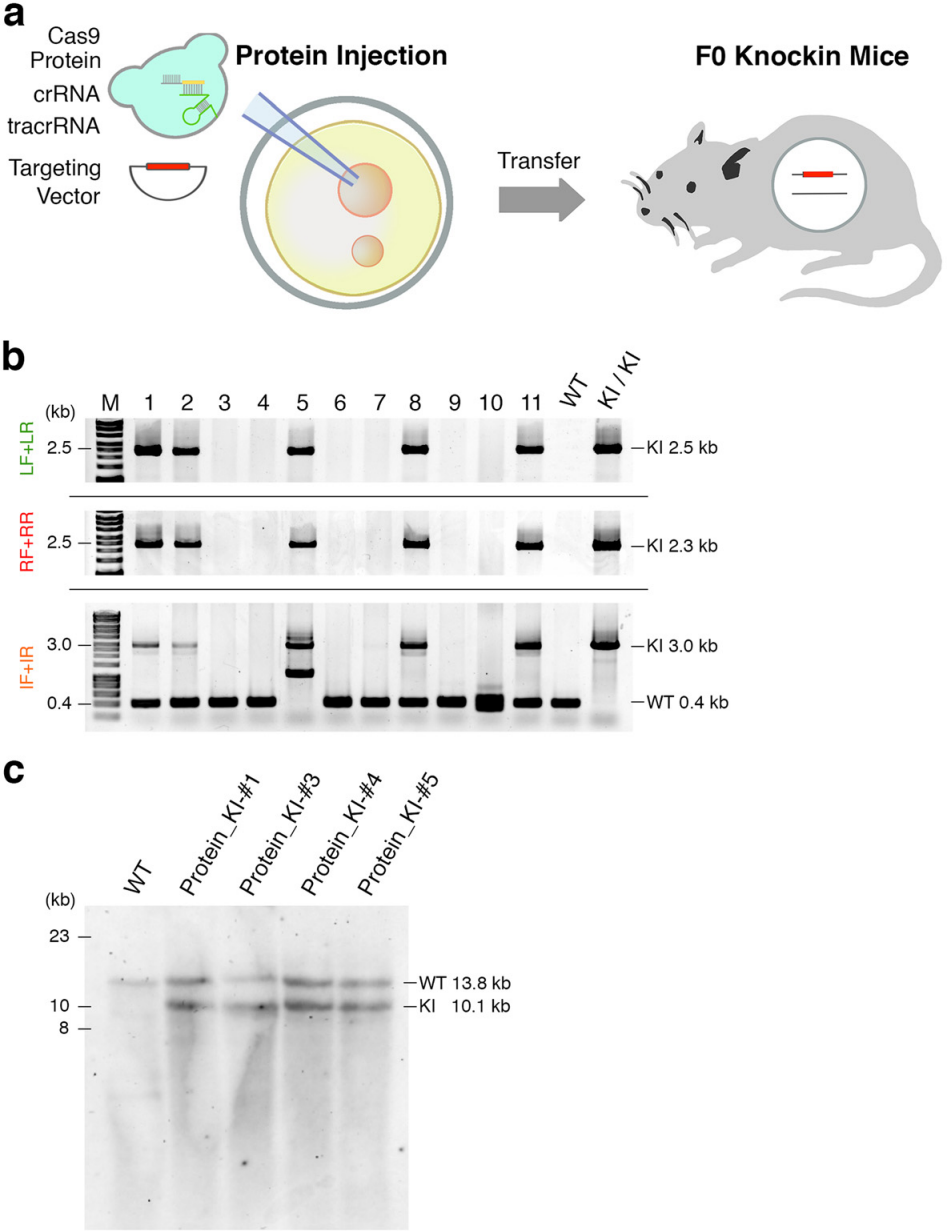
Highly efficient generation of knock-in mice carrying a gene cassette by the cloning-free CRISPR/Cas system. **(a)** Schematic diagram of pronuclear injection of Cas9 protein, chemically synthesized crRNA and tracrRNA and TetO-FLEX-EGFP-polyA targeting vector. **(b)** PCR screening of knock-in newborns derived from pronuclear protein injection. **(c)** Southern blotting of knock-in newborns derived from pronuclear protein injection. The knock-in mice, Protein_KI-#1, 3, 4, and 5, correspond to the newborn mice 1, 5, 8, and 11 in **(b)**, respectively. IF, internal forward primer; IR, internal reverse primer; KI, knock-in; LF, left forward primer; LR, left reverse primer; RF, right forward primer; RR, right reverse primer; M, molecular marker; WT, wild type.

Next, we tested whether the knock-in efficiency was improved by the cloning-free CRISPR/Cas system. We first injected a mixture of 30 ng/μl Cas9 protein, 0.061 pmol/μl *Actb* crRNA, 0.061 pmol/μl tracrRNA, and 10 ng/μl targeting vector into the pronuclei of zygotes (Figure 4a). The molar concentrations of dual RNAs were equivalent to that of sgRNA in Figures 1 and 2. We obtained nine newborn mice, but none carried the TetO-FLEX-EGFP-polyA cassette at the *Actb* locus (Figure S8 and Table S3 in Additional file 1), consistent with the low digest activity at this concentration shown by IDA *in vitro* (Figure 3b), and the endogenous *Actb* loci were targeted by NHEJ in three out of eight mice (Figure S9 and Table S3 in Additional file 1). Next, we injected a mixture containing a higher dose of the dual RNAs (30 ng/μl Cas9 protein, 0.61 pmol/μl *Actb* crRNA, 0.61 pmol/μl tracrRNA, and 10 ng/μl targeting vector) into the pronulei of zygotes. Surprisingly, we found 5 correctly targeted knock-in mice among 11 newborn mice (Figure 4b). The efficiency of the targeted insertion of the transgene by Cas9 protein injection combined with a higher dose of chemically synthesized crRNA and tracrRNA was 45.5% (Table 1). We further confirmed genotypes by Southern blotting with genomic DNA of knock-in newborns (Figure 4c). We also found the endogenous *Actb* loci were targeted by NHEJ in four out of six non-knock-in mice (Table 1; Figure S10 and Table S3 in Additional file 1). These results suggest that the direct pronuclear delivery of the Cas9 protein-chemically synthesized dual RNAs complex majorly facilitates the generation of knock-in mice carrying a functional gene cassette.

Because all the knock-in mice we generated were heterozygous for the TetO-FLEX-EGFP-polyA cassette at the *Actb* loci (Figures 1c and 4b,c), we investigated damage to non-knock-in alleles of these knock-in mice. We cloned a 400 bp band of PCR product amplified with internal forward and internal reverse primers, and sequenced them. Four out of six knock-in mice showed damage to non-knock-in alleles, including small deletions and long insertions (Figure S11 in Additional file 1). Interestingly, we found that one knock-in mouse (Protein_KI-#3) carried a 749 bp insertion corresponding to a part of *Trim33* 'mRNA' which expands on three exons at chromosome 3 whereas *Actb* is located on chromosome 5. These results suggest that non-knock-in alleles were damaged in about half of the knock-in mice generated by Cas9 protein combined with dual RNAs.

We further investigated off-target cleavage in the knock-in mice, which is the most serious problem [24-27] associated with CRISPR/Cas-mediated genome editing. We chose 14 off-target candidate loci (OT1-14) containing up to 3 bp mismatches compared with the 20 bp guide sequence of *Actb* crRNA [18,25]. Among 13 off-target candidate loci (OT6 could not be amplified by PCR) in 6 knock-in mice, we did not find any sign of off-target digestion (Figure S12 and Table S4 in Additional file 1). These results suggest that *in vivo* genome editing by Cas9 protein combined with dual RNAs is highly specific for the on-target locus.

Finally, we crossed F0 knock-in mice with wild-type mice to investigate germline transmission of the knock-in alleles to the F1 generation. We tested four knock-in lines using PCR and Southern blotting and found that all showed successful germline transmission in F1 progeny with an average efficiency of 51.4% (ranging from 37.5% to 61.1% between lines) (Figures S13 and S14, and Table S5 in Additional file 1). These results suggest that the percentage of mosaicism in F0 knock-in mice was very low.

### Functionality of the reporter cassette inserted at the *Actb* locus

**Figure 5:**
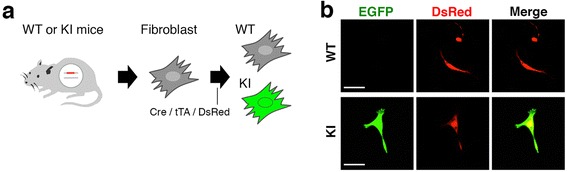
Functionality of the reporter cassette inserted at the *Actb* locus. **(a)** Schematic diagram of primary fibroblast cultures and transfection of three plasmids. **(b)** Confocal images of transfected fibroblasts derived from knock-in (KI) mice and their control littermates (wild type, WT).

Finally, we tested whether the TetO-FLEX-EGFP-polyA cassette inserted into the mouse *Actb* locus is effective. We transfected Cre-, tTA-, and DsRed-expressing plasmids into primary mouse fibroblasts derived from ear tips of three F0 knock-in and control littermates (Figure 5a). We found strong EGFP fluorescence only in fibroblasts derived from knock-in mice (Figure 5b). These results suggest that functional EGFP proteins are produced from the TetO-FLEX-EGFP-polyA cassette inserted into the endogenous *Actb* locus under the presence of Cre and tTA.

## Discussion

In this study, we developed a cloning-free CRISPR/Cas-mediated genome editing system for highly efficient and convenient one-step generation of knock-in mice carrying a functional gene cassette. This system has several advantages. First, the CRISPR/Cas vector construction and *in vitro* RNA transcription can be omitted by using commercially available Cas9 protein and chemically synthesized crRNA and tracrRNA, leading to a cloning-free CRISPR/Cas system. Although chemical synthesis of sgRNA might also be possible and convenient, technical limitations for the synthesis of long sgRNAs (more than 100 mer) must be considered. In contrast, shorter crRNAs and tracrRNAs can be chemically synthesized easily in a cost-effective manner. Furthermore, tracrRNAs can be commonly used independently of target sequences as well as Cas9 protein. The targeting vectors are already chemically synthesizable. Second, the efficiency of CRISPR/Cas-mediated digestion can be evaluated with a cell-free IDA system using target *Actb* PCR product, Cas9 protein, and chemically synthesized crRNA and tracrRNA, instead of cellular SSA or Cel-I assays. Third, and most importantly, the direct delivery of Cas9 protein, chemically synthesized crRNA and tracrRNA, and targeting vector into the pronuclei of zygotes allowed for the highly efficient generation of knock-in mice carrying gene cassettes in the endogenous gene. Since we could not obtain any knock-in mice by the direct delivery of Cas9 protein, sgRNA, and targeting vector into the pronuclei of zygotes, the high knock-in efficiency of the cloning-free CRISPR/Cas system was mediated by use of crRNA and tracrRNA, not conventional sgRNA. Although both sgRNA and dual RNAs combined with Cas9 protein efficiently digested the target *Actb* PCR product in a biochemical IDA (Figure 3c), the enzymatic reaction conditions were completely different from those of zygote injection, leading to discrepancy in NHEJ and HR efficiencies *in vivo*. Consistent with this idea, although the sgRNA co-injected with Cas9 mRNA showed high NHEJ efficiency regardless of targeting vector (Table 1; Table S1 in Additional file 1), the sgRNA combined with Cas9 protein showed much less NHEJ efficiency (Table 1; Table S2 in Additional file 1), suggesting non-optimal assembly of Cas9 protein and sgRNA to form a highly active ribonucleoprotein complex. Taken together, the cloning-free CRISPR/Cas system facilitates functional cassette knock-in into the mouse chromosome.

In contrast to the highly efficient (almost 100%) generation of NHEJ-mediated knockout mice by the injection of *Actb* sgRNA and Cas9 mRNA, we demonstrated much lower efficiency of HR-mediated generation of knock-in mice by the injection of these RNAs combined with targeting vector. One possible explanation for this is the difference in the injection method. For knockout mice production, we injected *Actb* sgRNA and Cas9 mRNA into the cytoplasm, which presumably led to fast translation of Cas9 mRNA into protein, as described previously [5,6]. For knock-in mice production, because pronuclear injection [5,7-9] is a standard method for delivering RNA and targeting vector into one-cell-stage mouse zygotes, we injected the mixture of *Actb* sgRNA, Cas9 mRNA and targeting plasmid vector into the pronuclei, which might lead to delays in the translation of Cas9 mRNA into protein.

Rapid digestion of target genomic DNA within one-cell zygotes may also be critical for highly efficient induction of HR. In a previous cellular study, it was shown that the target genomic DNA was almost immediately digested by the direct delivery of the Cas9 protein-RNA complex [22]. Further, highly efficient generation of knockout mice by the direct delivery of the Cas9 protein-RNA complex was reported [23]. Thus, direct injection of the Cas9 protein-RNA complex into pronuclei may result in immediate digestion of the target endogenous locus of mouse zygotes at the early one-cell stage, leading to highly efficient knock-in of the functional cassette. Consistent with this view, knock-in alleles in four knock-in mice generated by injection of a Cas9 protein-dual RNAs complex were successfully transmitted to F1 progeny with about 50% efficiency.

It was also reported that a Cas9 protein-RNA complex was rapidly degraded in cultured cells, thus reducing undesired off-target effects [22,28], the most serious problem [24-27] associated with CRISPR/Cas-mediated genome editing. In contrast to high off-target effects in cultured cell lines [24-27], its frequency is thought to be relatively rare in embryonic stem cells and mouse embryos [9]. Our off-target analysis in several candidate loci in knock-in mice confirmed the high accuracy of the CRISPR/Cas system with the Cas9 protein-dual RNAs complex in mouse embryos. Although non-biased genome-wide off-target analysis [29-31] or whole-genome sequencing [32-34] will be required, use of the Cas9 protein-dual RNAs complex is a promising approach for highly specific genome editing. Thus, our cloning-free CRISPR/Cas-mediated *in vivo* genome editing system provides highly efficient and highly accurate generation of knock-in mice carrying functional cassettes.

The insertion of several hundred nucleotides is often found at the targeted loci when using genome editing, although the origin of the inserted sequence is unknown [35]. One possible source of the insertion is the transcribed RNA. Recent studies suggest that DNA double-strand breaks (DSBs) are repaired by using the DNA that is reverse-transcribed from mRNA in mammalian cells [36], yeast [37], and fly [38]. We found trans-insertion of *Trim33* mRNA into the *Actb* locus in our knock-in mouse, providing direct evidence of transcript RNA-templated DNA DSB repair in mammalian organisms *in vivo*. Transcript RNA-templated repair of DNA DSBs is mutagenic and polymorphic in the human genome [36], suggesting that it may play an important role in human genetic diseases and evolution. Thus, our results shed light on transcript RNA-templated DNA DSB repair in mammalian organisms *in vivo* and stimulate functional research on this.

In addition to the generation of knock-in mice carrying complex gene cassettes, our method can be directly applied to the generation of knockout mice [23] and knock-in mice carrying single nucleotide substitutions with oligo DNA donors [5,7,8], as well as to other species [39-41] and cultured cells [22,28], and *in vivo* genome editing in adult animals [42]. Taken together, our streamlined cloning-free CRISPR/Cas-mediated *in vivo* genome editing system enables the highly efficient and extremely convenient one-step generation of knock-in mice carrying functional gene cassettes.

## Conclusions

Cas9 protein and chemically synthesized crRNA and tracrRNA enabled cloning-free CRISPR/Cas system without CRISPR vector construction, cellular experiments for evaluation of the digestion activity of CRISPR/Cas, and *in vitro* RNA transcription. By the direct nuclear delivery of Cas9 protein complex combined with dual RNAs into one-cell mouse zygotes, knock-in mice carrying functional cassette were generated with extreme high efficiency, which could not be achieved by conventional mRNA pronuclear injection or Cas9 protein injection combined with commonly used sgRNA. Taken together, our streamlined cloning-free CRISPR/Cas-mediated *in vivo* genome editing system provides the highly efficient and extremely convenient one-step generation of knockout and knock-in animals, leading to acceleration of *in vivo* functional genomic research.

## Materials and methods

### Animal experiments

All research and animal care procedures were approved by the Tokyo Medical and Dental University Animal Care and Use Committee. Mice were housed in groups of three to five animals per cage and maintained on a regular 12 hours light/dark cycle (8:00 to 20:00 light period) at a constant 25°C. Food and water were available *ad libitum*.

### CRISPR/Cas plasmid

A pair of oligo DNAs (Hokkaido Systems Science, Sapporo, Hokkaido, Japan) corresponding to *Actb* sgRNA was hybridized and ligated using Quick Ligase (New England BioLabs (NEB), Ipswich, MA, USA) into linearized pX330 plasmid (Addgene, 42230; Feng Zhang, MIT) digested with BbsI (NEB) as previously described [18,19]. Oligo DNAs and primers are listed in Table S6 in Additional file 1.

### Targeting vector

The p*Actb*-TetO-FLEX-EGFP-polyA targeting vector was constructed based on a pAAV-TetO-FLEX-HA-mKate2-TeNT-polyA plasmid (a gift from Akihiro Yamanaka, Nagoya University) with several modifications. First, HA-mKate2-TeNT was excised by digestion with XhoI (NEB) and HindIII (NEB), and replaced with PCR amplified inverted EGFP. Second, AAV2-ITR was excised by digestion with NarI (NEB) and BstEII (NEB), and replaced with a PCR-amplified 2.0 kb *Actb* fragment from C57BL/6 J mouse genomic DNA for left homology arm using a In-Fusion HD Cloning Kit (Takara, Otsu, Shiga, Japan). Finally, the PCR-amplified 2.0 kb *Actb* fragment for right homology arm was inserted into the plasmid digested with NotI (NEB) and MluI (NEB) by In-Fusion reaction.

### Single-strand annealing assay

SSA assay using HEK293T cells was performed as described previously [43]. Briefly, *Actb*-pX330 or empty pX330 plasmids, firefly luciferase reporter vector containing the PCR-amplified *Actb* target sequence (Table S6 in Additional file 1), and renilla luciferase-expressing reference vector were co-transfected into HEK293T cells in a 96-well plate using Lipofectamine LTX (Life Technologies, Grand Island, NY, USA). At 24 hours post-transfection, luciferase activity was measured using the Dual-Glo Luciferase Assay System (Promega, Madison, WI, USA) according to the manufacturer’s instructions.

### Cel-I assay in Neuro2A cells

Cel-I assay using Mouse Neuro2A cells was performed as described previously [43]. Briefly, *Actb*-pX330 or empty pX330 plasmids was transfected into Neuro2A cells in a 6-well plate using Lipofectamine LTX (Life Technologies). After 72 hours post-transfection, genomic DNA was isolated using a DNeasy Blood & Tissue Kit (Qiagen, Valencia, CA, USA). Then, *Actb* loci were PCR amplified from the purified genomic DNA with primers (Table S6 in Additional file 1). PCR products were denatured, digested at 42°C for 30 minutes with a Surveyor Mutation Detection Kit (Transgenomic, Omaha, NE, USA), and analyzed by electrophoresis in 3% agarose gel stained with ethidium bromide. Gel images were obtained with a ChemiDoc XRS system (Bio-Rad, Hercules, CA, USA) and analyzed by Image Lab software (Bio-Rad).

### *In vitro* RNA transcription

Cas9 mRNA and *Actb*-sgRNA were prepared as described previously [5]. Cas9 and *Actb*-sgRNA were PCR amplified from *Actb*-pX330 with T7 promoter-attached primers (Table S6 in Additional file 1). T7-Cas9 and T7-*Actb*-sgRNA PCR products were purified with a PCR Purification Kit (Qiagen) and used as the template for *in vitro* transcription using a mMESSAGE mMACHINE T7 ULTRA Kit (Life Technologies) and MEGAshortscript T7 Kit (Life Technologies). Cas9 mRNA and *Actb*-sgRNA were purified with a MEGAclear Kit (Life Technologies) and eluted with Nuclease-free water (Life Technologies). The quality of RNAs was analyzed using a NanoDrop (Thermo Scientific, Waltham, MA, USA) and Bioanalyzer (Agilent Technologies, Santa Clara, CA, USA).

### Cas9 proteins

The recombinant Cas9 proteins were obtained from NEB and PNA Bio (Thousand Oaks, CA, USA).

### Chemical synthesis of crRNA and tracrRNA

*Actb*-crRNA (5′-cauuaugaguccuuaagugaGUUUUAGAGCUAUGCUGUUUUG-3′) and -tracrRNA (5′- AAACAGCAUAGCAAGUUAAAAUAAGGCUAGUCCGUUAUCAACUUGAAAAAGUGGCACCGAGUCGGUGCU-3′) were designed with some modification of previously reported methods [12,19], and chemically synthesized and purified by polyacrylamide gel electrophoresis (Fasmac, Atsugi, Kanagawa, Japan).

### *In vitro* digestion assay

Cas9 proteins (30 ng/μl) and chemically synthesized *Actb*-crRNA (8.7 ng/μl) and -tracrRNA (14.3 ng/μl), or *in vitro* transcribed *Actb*-sgRNA (25 ng/μl) were incubated with *Actb* target PCR products (30 ng/μl) in a Cas9 Nuclease Reaction Buffer (NEB) at 37°C for 60 minutes as previously described [13], then treated with RNase A (5 mg) and incubated at 37°C for 30 minutes to remove RNA [44]. Reactions were stopped with 6× DNA loading buffer containing 30% glycerol, 1.2% SDS and 250 mM EDTA, and analyzed by electrophoresis in 2% agarose gel as described above.

### Injection

For knockout mouse production, Cas9 mRNAs and *Actb*-sgRNA were diluted and mixed in 0.1 TE buffer (10 mM Tris-HCl, 0.1 mM EDTA (pH 8.0)) to a working concentration of 50 and 20 ng/μl, respectively. One-cell-stage zygotes were obtained by mating of BDF1 males and females (CLEA Japan, Meguro, Tokyo, Japan), and then frozen and stored until use. The mixture of Cas9 mRNAs and *Actb*-sgRNA was injected into the cytoplasm using a micromanipulator and microscope (Leica, Wetzlar, Germany) and injector (Eppendorf, Hauppauge, NY, USA). After incubation at 37°C for 24 hours, two-cell-stage embryos were transferred into pseudopregnant ICR female mice (CLEA Japan).

For knock-in mouse production by RNA injection, Cas9 mRNAs, *Actb*-sgRNA, and p*Actb*-TetO-FLEX-EGFP-polyA were diluted and mixed in 0.1 TE buffer to a working concentration of 5, 2.5, and 10 ng/μl, respectively, as previously described [5]. The injection mixture was injected into pronuclei of one-cell-stage zygotes.

For knock-in mouse production by protein injection, Cas9 proteins, *Actb*-sgRNA, or *Actb*-crRNA and tracrRNA, and p*Actb*-TetO-FLEX-EGFP-polyA were diluted and mixed in 0.1 TE buffer to a working concentration of 30 or 100 ng/μl, 2.5 or 25 ng/μl, or 0.061 or 0.61 pmol/μl, and 10 ng/μl, respectively. The mixture was incubated at 37°C for at least 15 minutes, and then injected into pronuclei of one-cell-stage zygotes.

### PCR screening

Genomic DNA was prepared from F0 and F1 newborn tails by proteinase K treatment and a subsequent standard phenol extraction method as described previously [45,46]. Knock-in mice were screened by PCR with ExTaq (Takara) and three different pairs of primers (Table S6 in Additional file 1) and analyzed by electrophoresis in 1 or 2% agarose gel as described above. PCR products were further cloned with TOPO TA Cloning Kit (Life Technologies) and analyzed by sequencing.

### Southern blotting

Southern probe (0.7 kb) was PCR amplified (Primers: Table S6 in Additional file 1) from BDF1 genomic DNA and cloned with a TOPO TA Cloning Kit, and DIG-labeled with a DIG-High Prime DNA Labeling and Detection Starter Kit II (Roche, Penzberg, Upper Bavaria, Germany). The genomic DNA was digested with HindIII and separated on 0.7% agarose gel, transferred to nylon membranes, positively charged (Roche), hybridized with DIG-labeled probe, and detected with CDP-Star (Roche) as previously described [47].

### Off-target effects

The potential off-target candidate loci containing up to 3 bp mismatches compared with the 20 bp guide sequence of *Actb* sgRNA were predicted by the CRISPR design tool [18,25,48]. The off-target candidate loci were amplified by PCR using primers listed in Table S6 in Additional file 1 and analyzed by direct sequencing as previously described [47].

### Primary fibroblast cultures

The ear tips derived from 2-week-old mice were diced into small pieces, incubated at 37°C for 30 minutes with 4 mg/ml collagenase L (Nitta Gelatin, Naniwa, Osaka, Japan) and 4 mg/ml dispase (Life Technologies), and then cultured with 10% fetal bovine serum/Dulbecco’s modified eagle medium at 37°C and 10% CO_2_ for several days. pCAG-Cre, pCMV-tTA (Takara), and pCMV-DsRed (Takara) were co-transfected into primary fibroblast cells in a six-well plate using Lipofectamine LTX with Plus reagent (Life Technologies). Images were acquired on a FV500 confocal microscope and Fluoview software (Olympus, Shinjuku, Tokyo, Japan).

### Statistical analyses

All data are presented as the mean ± standard error of the mean. Statistical methods were described in the figure legends for each data set. Briefly, Student’s *t*-tests were used to compare differences between any two groups. One-way ANOVA with *post hoc* Tukey-Kramer tests were used to compare differences between three groups. Statistical significance was set at *P* < 0.05.

## Additional file

The following additional data are available with the online version of this paper. Additional file 1:
**Cellular assays, generation of knockout and knock-in** **mice, sequencing of all the newborns and** **non-knock-in** **alleles, germline transmission,** **off-target** **analysis, and tables of these results and a list of the oligo DNAs and primers used in this study.**

## Abbreviations

bp: base pair
Cas: CRISPR-associated protein
CMV: cytomegalovirus
CRISPR: clustered regularly interspaced short palindromic repeat
crRNA: CRISPR-RNA
DSB: double-strand break
EGFP: enhanced green fluorescent protein
FLEX: FLEX switch flanked by two pairs of loxP and lox2722
HR: homologous recombination
IDA: *in vitro* digestion assay
ITR: inverted terminal repeat
NEB: New England BioLabs
NHEJ: non-homologous end-joining
PCR: polymerase chain reaction
sgRNA: single guide RNA
SSA: single-strand annealing
tetO: Tet operator
tracrRNA: trans-activating crRNA
tTA: tetracycline transactivator
