# Supplementary material for: Cloning-free CRISPR/Cas system facilitates functional cassette knock-in in mice
Source: Genome Biol. 2015 Apr 29;16(1):87. doi: 10.1186/s13059-015-0653-x (PMC4414275; doi:10.1186/s13059-015-0653-x)
Supplement: Additional file 1: — Cellular assays, generation of knockout and knock-in mice, sequencing of all the newborns and non-knock-in alleles, germline transmission, off-target analysis, and tables of these results and a list of the oligo DNAs and primers used in this study. [file 13059_2015_653_MOESM1_ESM.docx]

**Additional file 1**

**Cloning-free CRISPR/Cas system facilitates functional cassette knockin in mice**

Tomomi Aida^1^, Keiho Chiyo^1^, Takako Usami^2^, Harumi Ishikubo^1^, Risa Imahashi^1^, Yusaku Wada^6^, Kenji F. Tanaka^5^, Tetsushi Sakuma^4^, Takashi Yamamoto^4^ & Kohichi Tanaka^1,3,7,§^

**
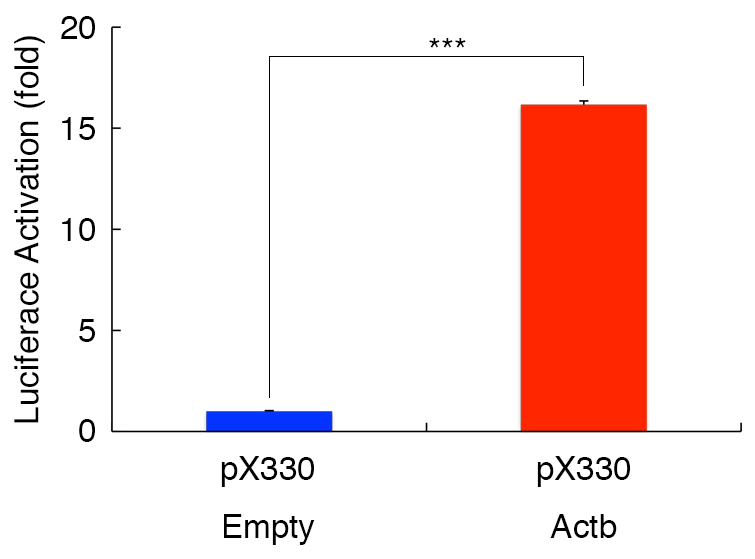
**

**Figure S1** Single-strand annealing assay using luciferase-based episomal plasmid vectors in HEK293T cells (n = 3 wells, respectively). Statistical significance was tested by t-test. ***p < 0.005.


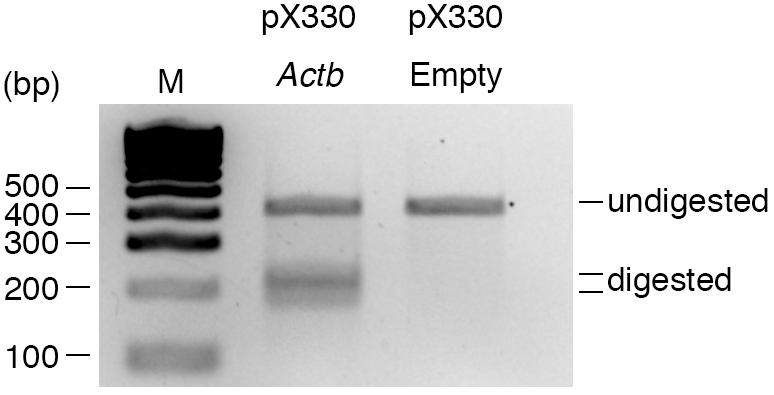


**Figure S2** Cel-I assay in mouse Neuro2A cells. M: molecular marker.


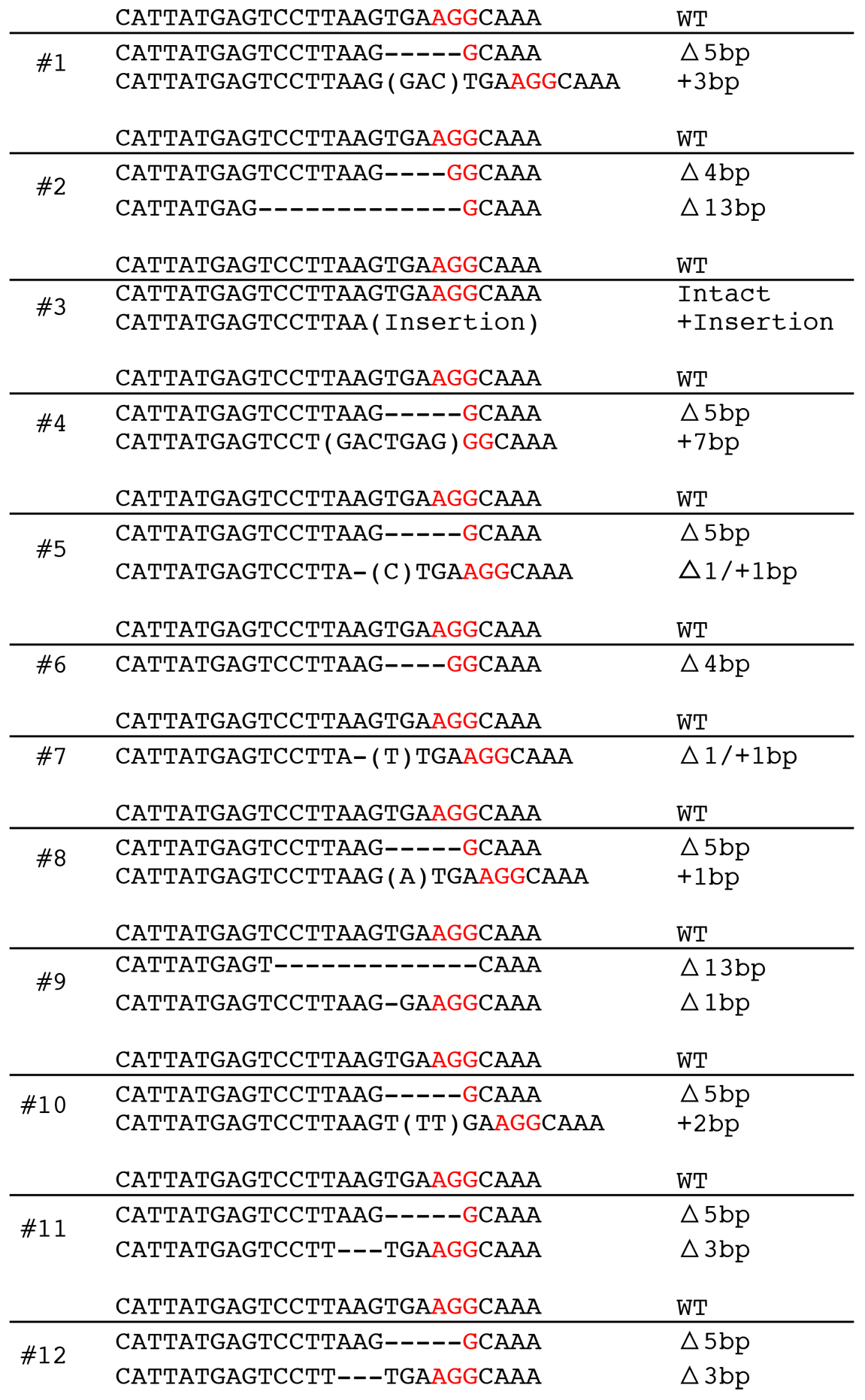


**Figure S3** The sequence of both alleles of *Actb* locus in the 12 newborn mice generated by injection of Cas9 mRNA and *Actb* sgRNA. PAM sequences are labeled in red.

**
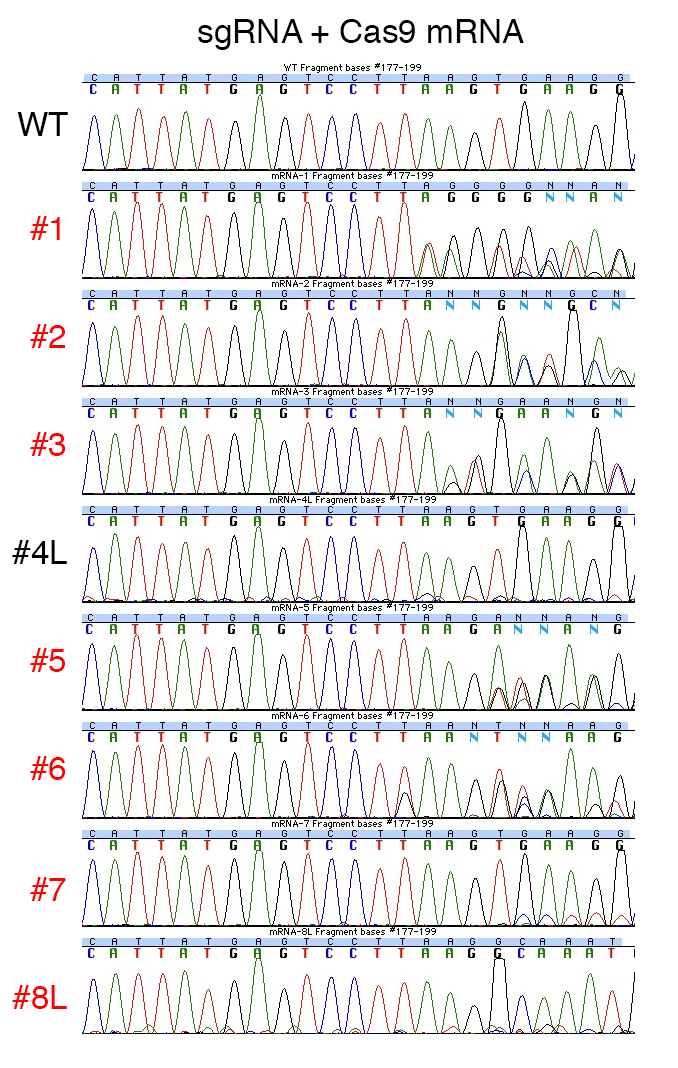
**

**
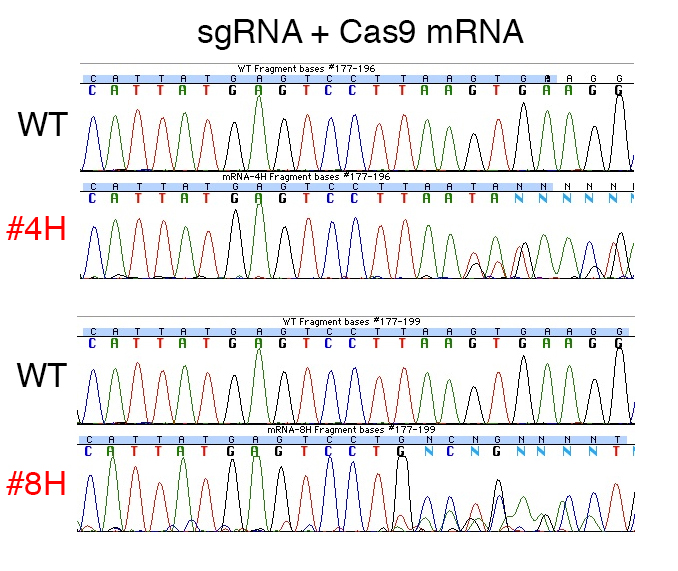
**

**Figure S4** Sequence analysis of the *Actb* loci in newborn mice generated by injection of Cas9 mRNA, *Actb* sgRNA and reporter construct, and a wildtype control (WT). Sequences were aligned to a common WT sequence (common in **Figures S4, 6-7,** and **9-10** in **Additional file 1**). The 20 bp target sequences and PAM are shown. Red: Modified mice. L and H: lower and higher bands.


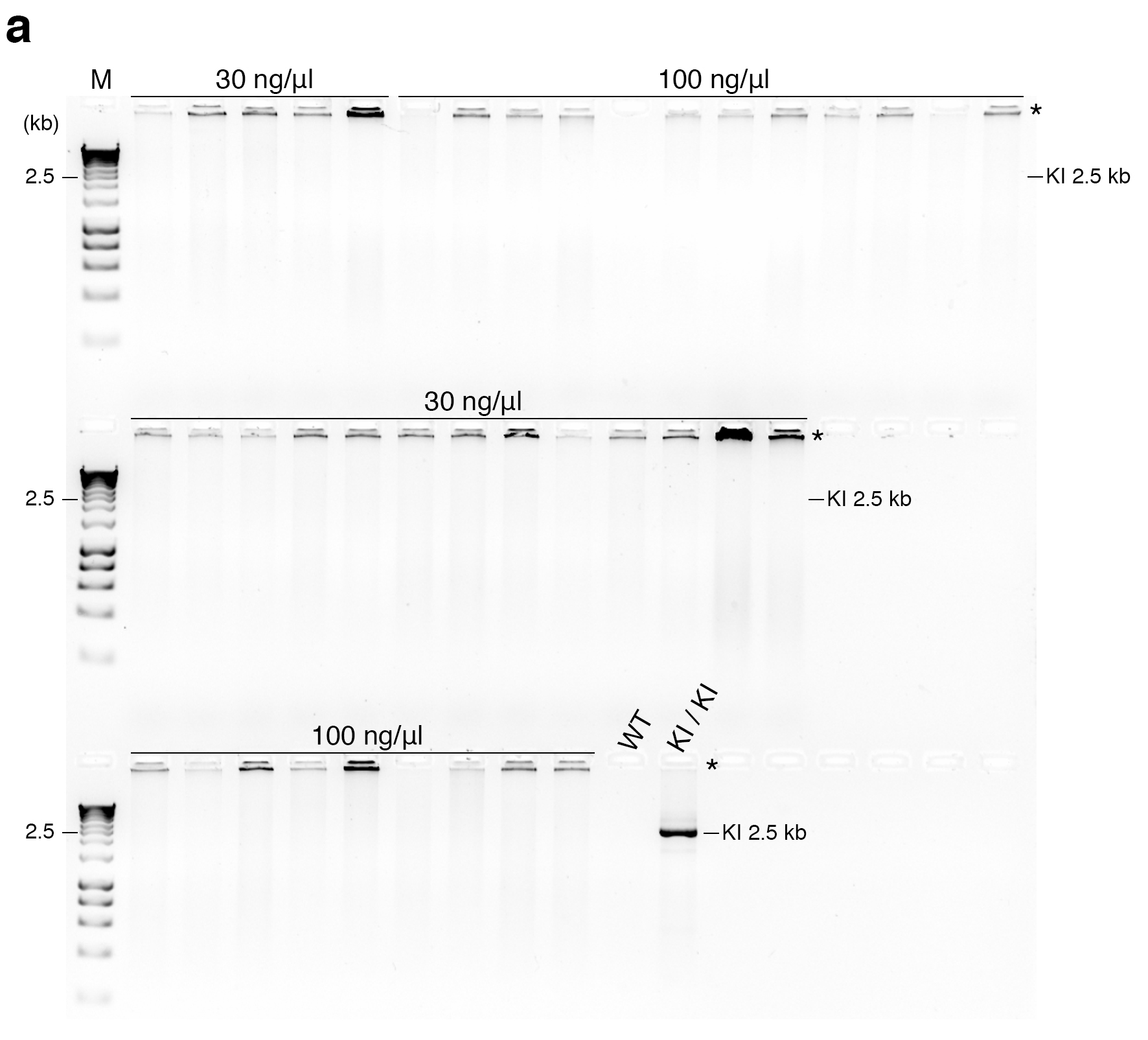


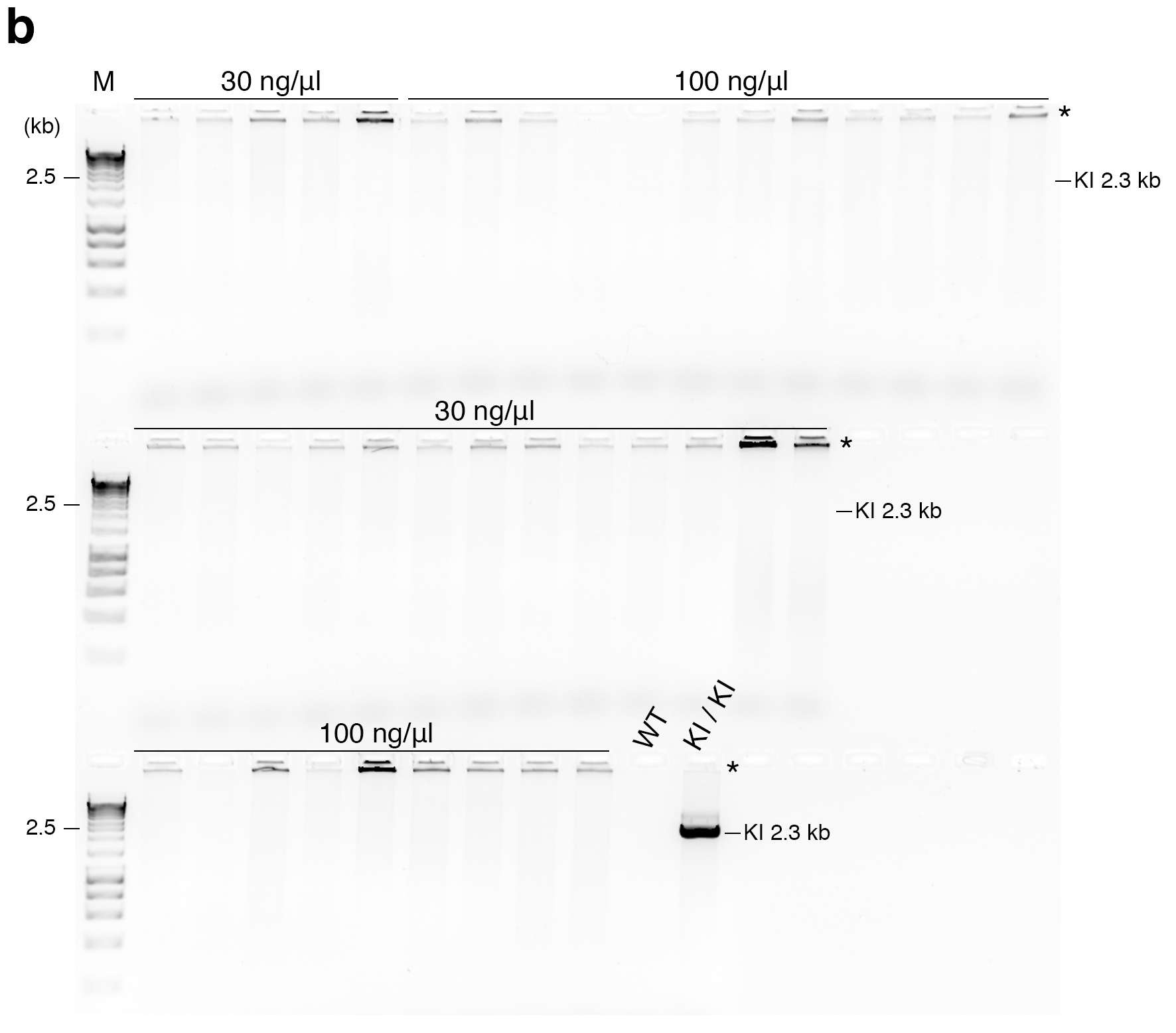


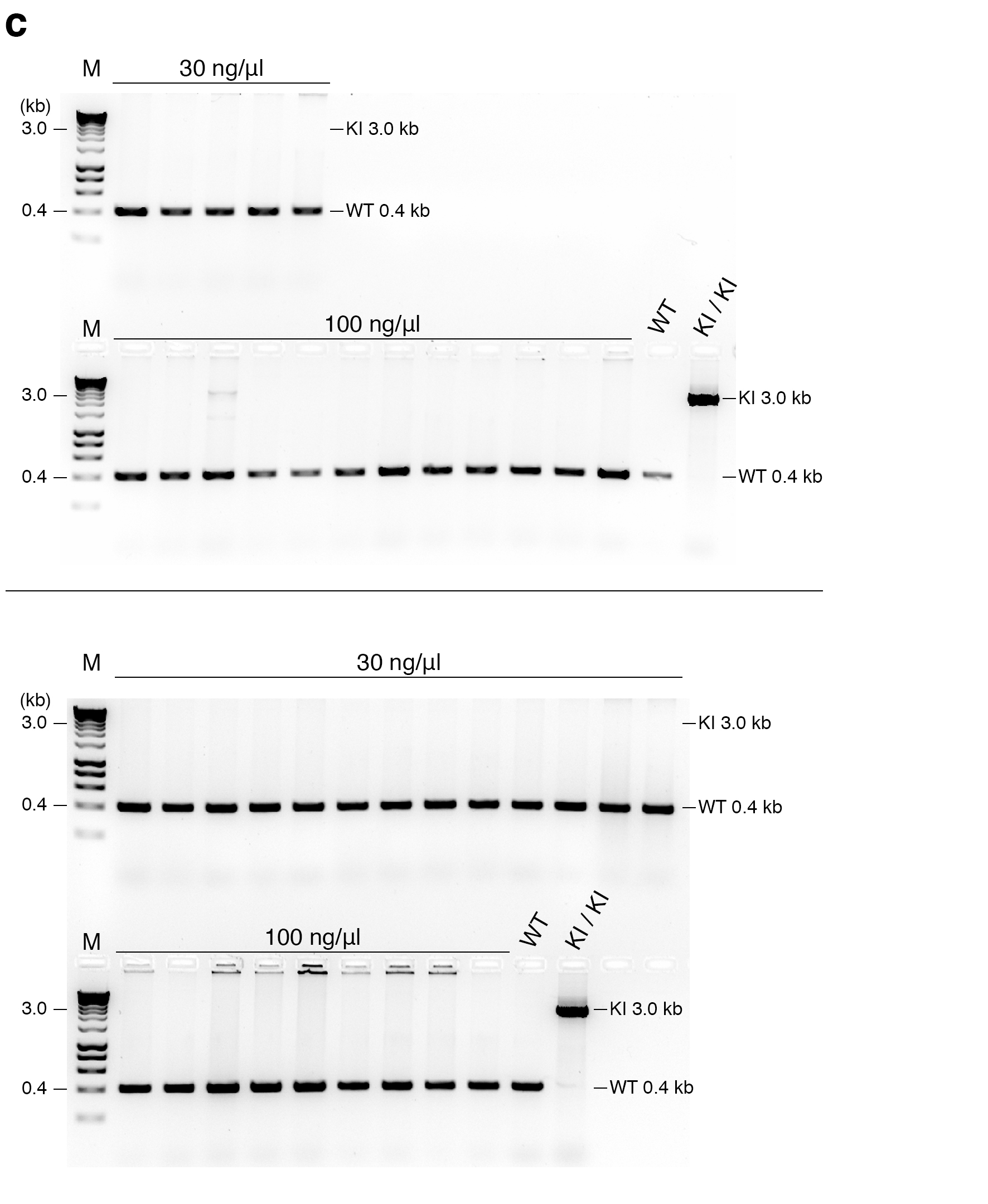
**Figure S5** PCR screenings of knockin newborns generated by coinjection of Cas9 protein, normal doses of sgRNA, and reporter construct. (**a-c**) Gel images of PCR products amplified with (**a**) LF+LR, (**b**) RF+RR, and (**c**) IF+IR primers. The concentrations of Cas9 protein are indicated above the horizontal lines. IF: internal forward primer, IR: internal reverse primer, LF: left forward primer, LR: left reverse primer, RF: right forward primer, RR: right reverse primer, M: molecular marker, WT: wildtype, and KI: knockin. *Non-specific signals in the wells of agarose gels.


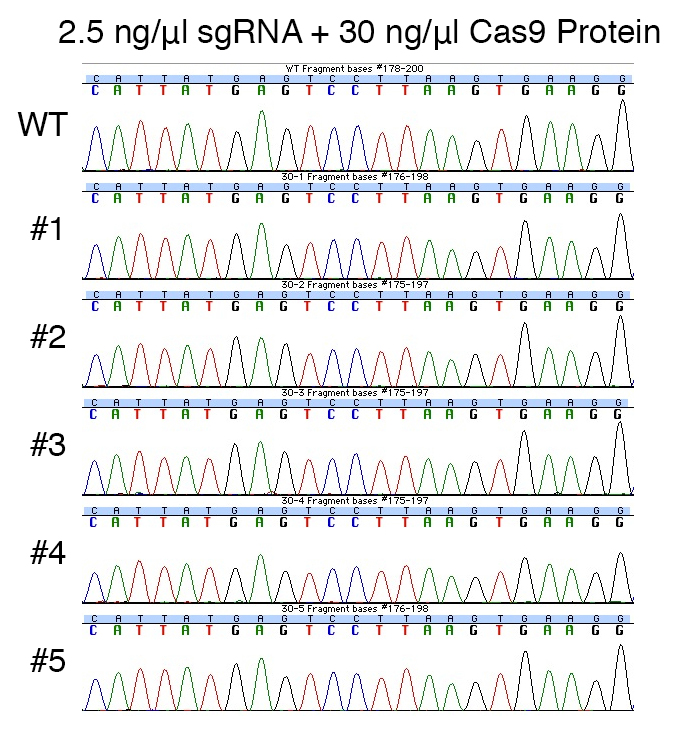

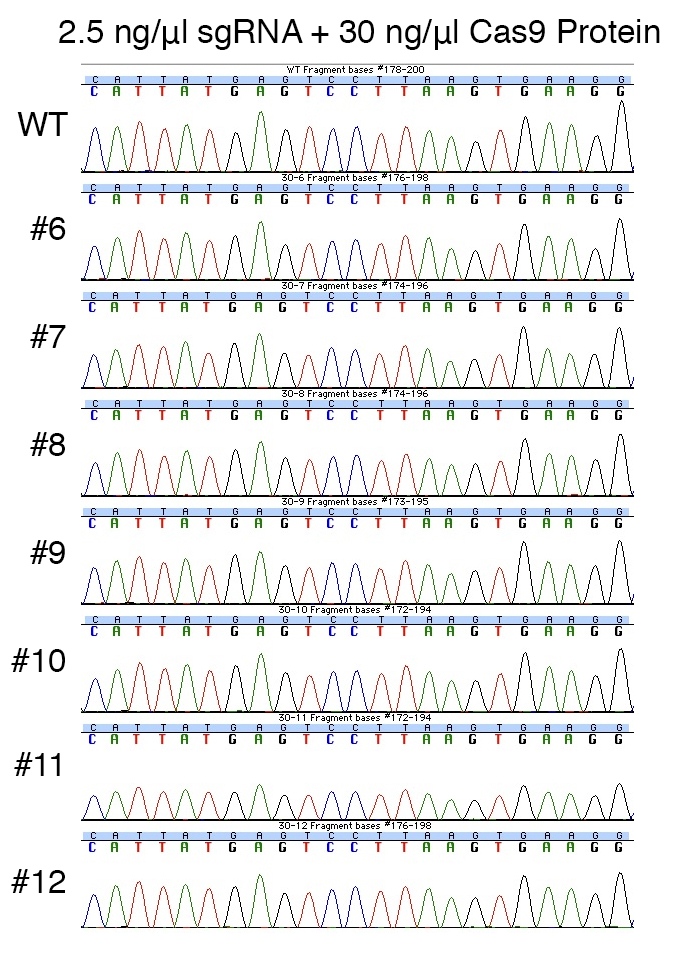


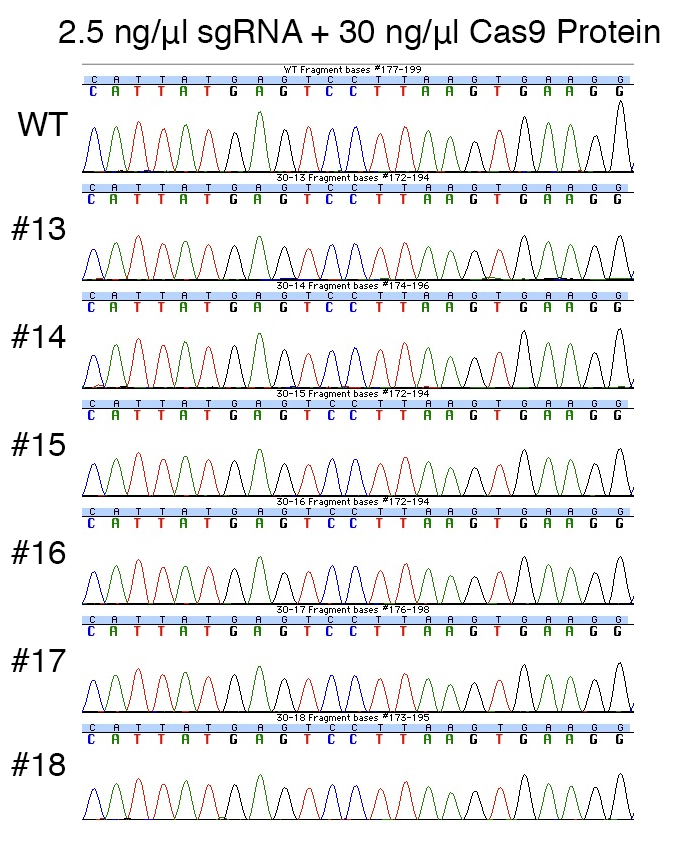


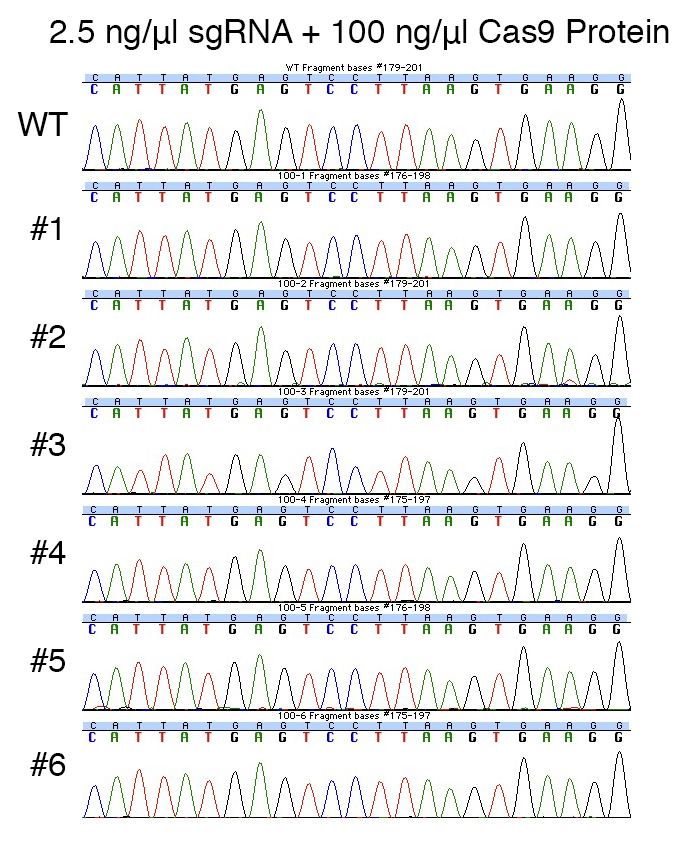


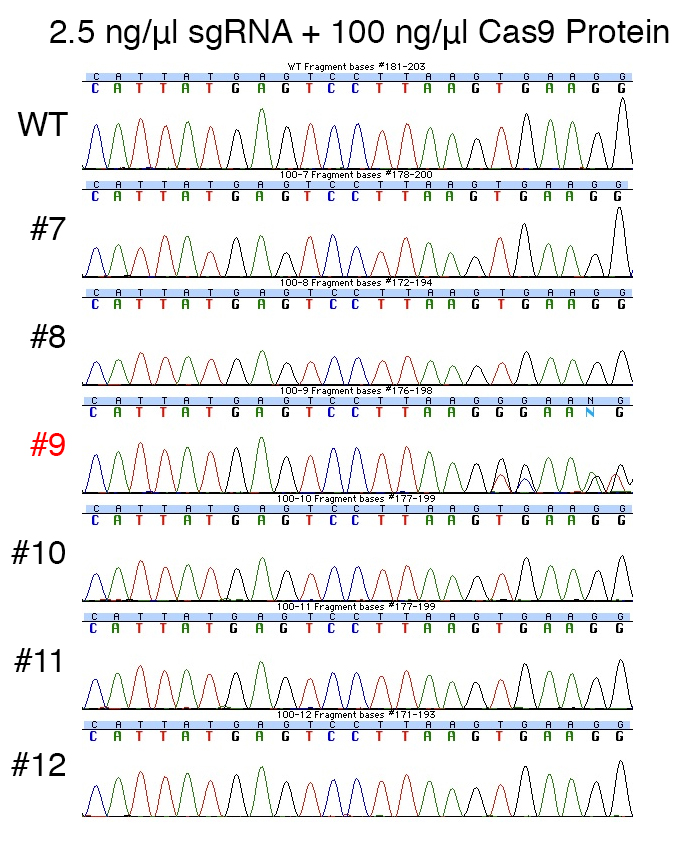


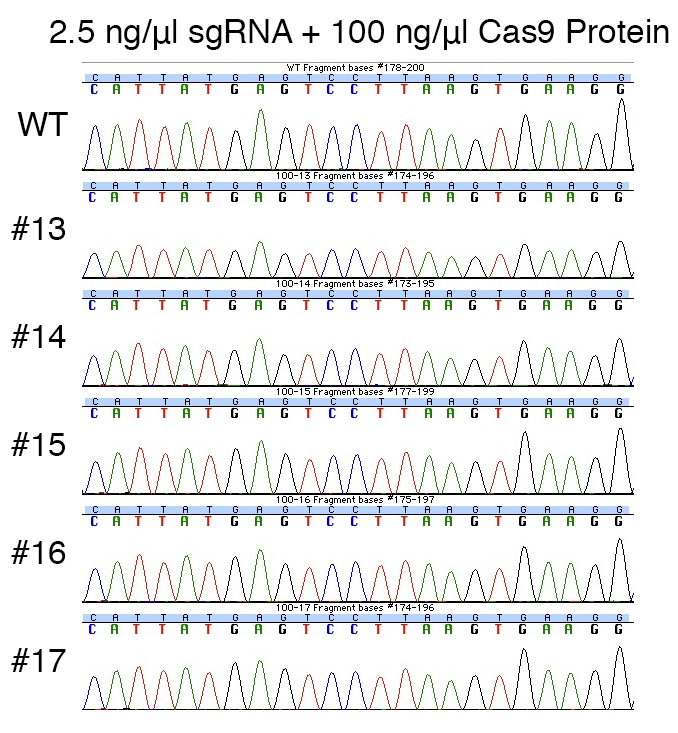


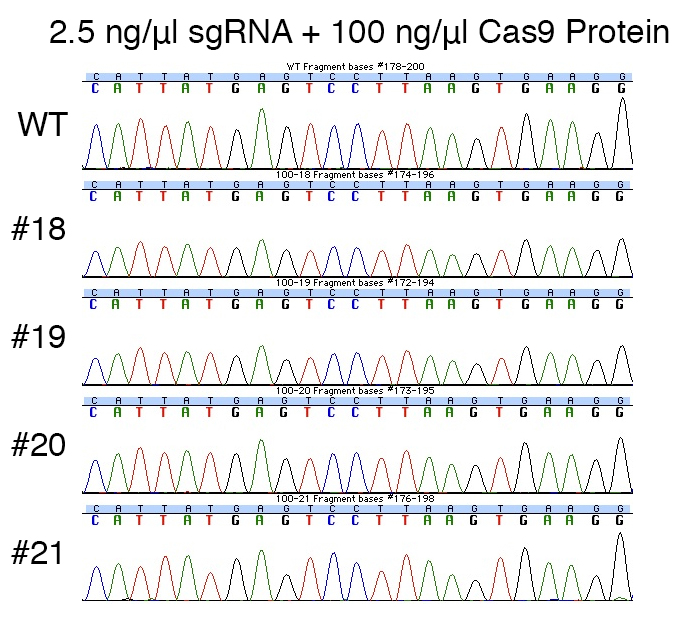


**Figure S6** Sequence analysis of the *Actb* loci in newborn mice generated by injection of Cas9 protein, normal doses of *Actb* sgRNA and reporter construct, and a wildtype control (WT). Sequences were aligned to a common WT sequence (common in **Figures S4, 6-7,** and **9-10** in **Additional file 1**). The 20 bp target sequences and PAM are shown. Red: Modified mice.


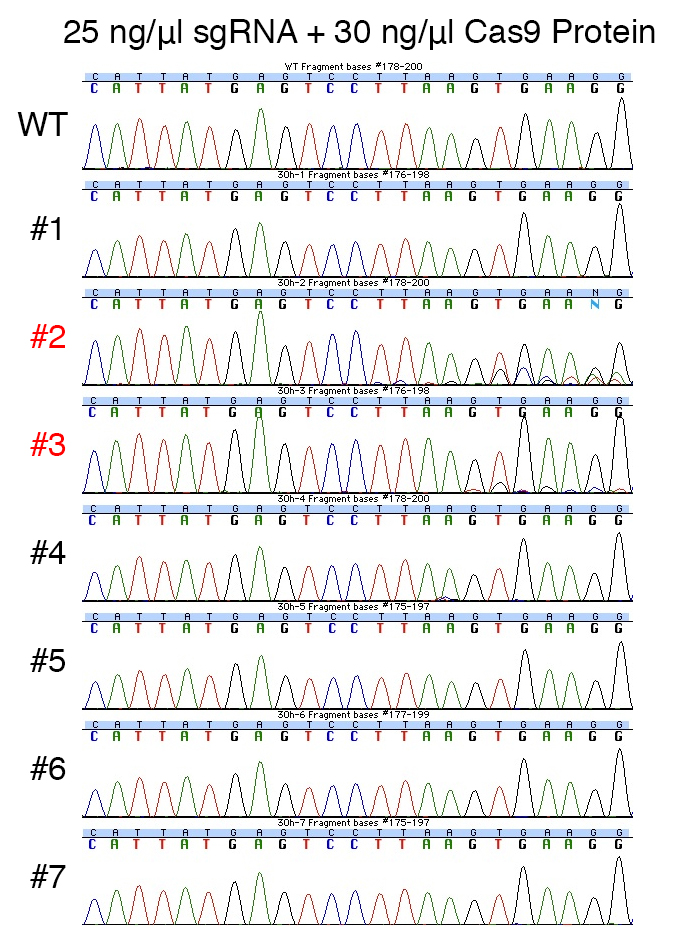


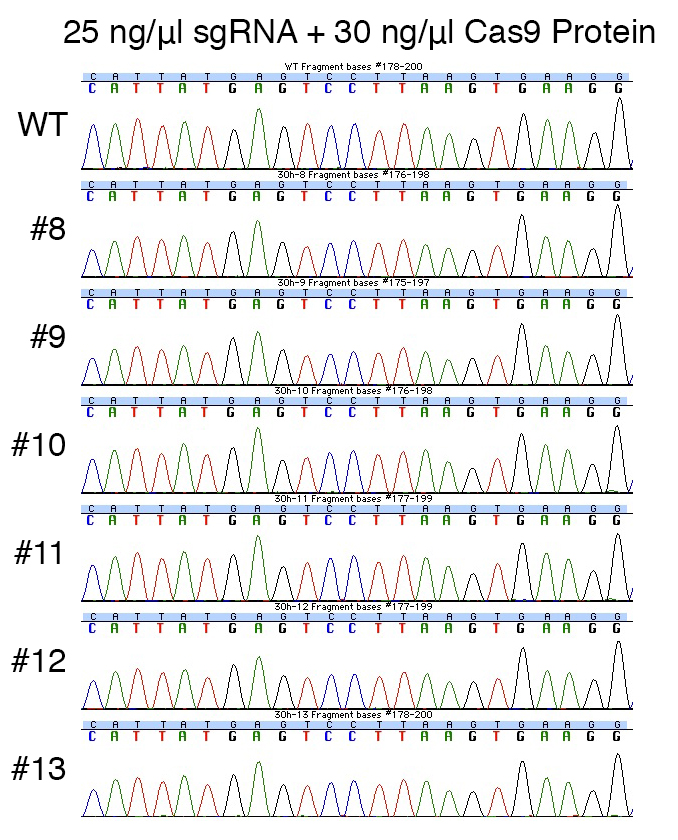


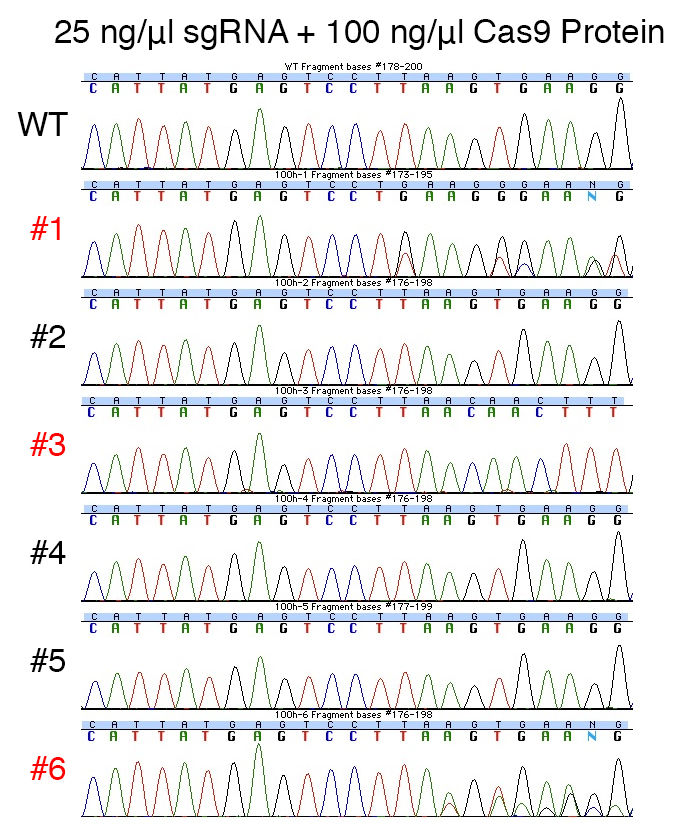


**Figure S7** Sequence analysis of the *Actb* loci in newborn mice generated by injection of Cas9 protein, higher doses of *Actb* sgRNA and reporter construct, and a wildtype control (WT). Sequences were aligned to a common WT sequence (common in **Figures S4, 6-7,** and **9-10** in **Additional file 1**). The 20 bp target sequences and PAM are shown. Red: Modified mice. Note that #3 (corresponds to #3 in Figure 2c) had homozygous transposon insertion.


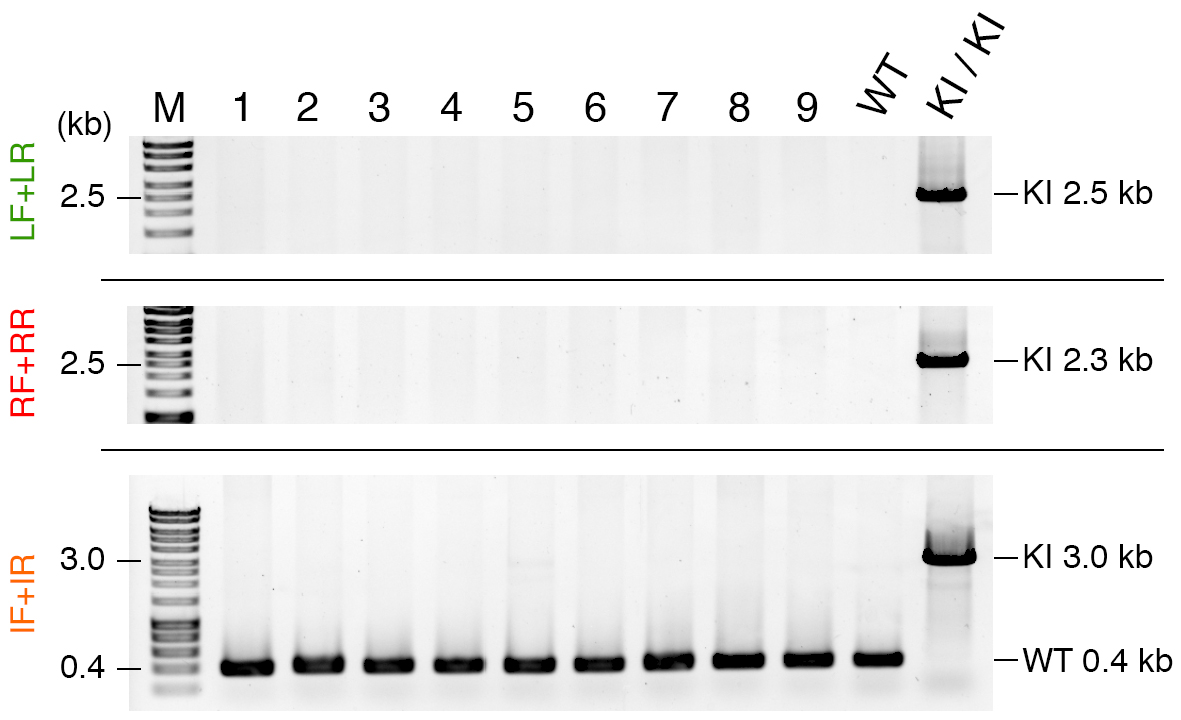


**Figure S8** PCR screenings of knockin newborns generated by coinjection of Cas9 protein, 0.061 pmol/μl crRNA and tracrRNA, and reporter construct. IF: internal forward primer, IR: internal reverse primer, LF: left forward primer, LR: left reverse primer, RF: right forward primer, RR: right reverse primer, M: molecular marker, WT: wildtype, and KI: knockin.


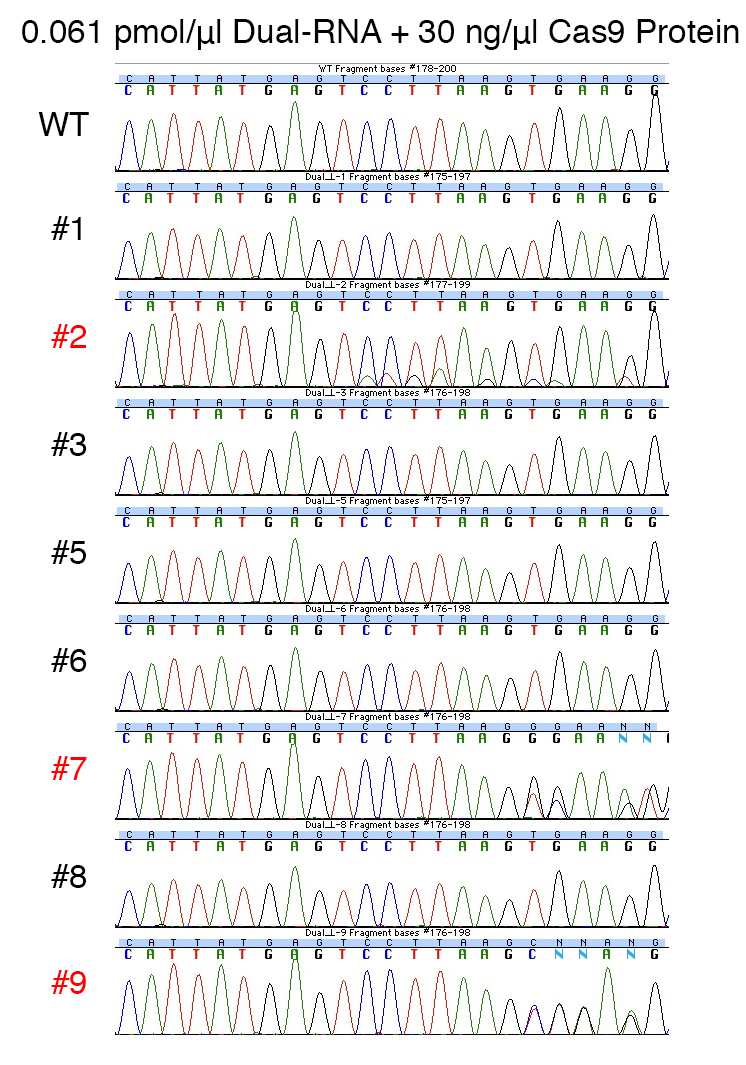


**Figure S9** Sequence analysis of the *Actb* loci in newborn mice generated by injection of Cas9 protein, 0.061 pmol/μl crRNA and tracrRNA and reporter construct, and a wildtype control (WT). Sequences were aligned to a common WT sequence (common in **Figures S4, 6-7,** and **9-10** in **Additional file 1**). The 20 bp target sequences and PAM are shown. Red: Modified mice.


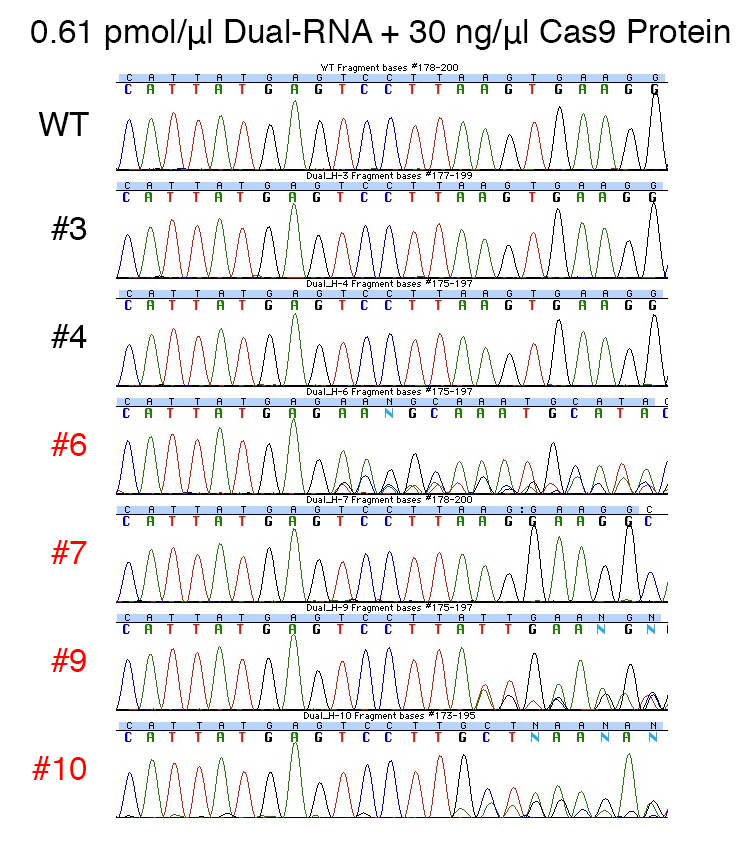


**Figure S10** Sequence analysis of the *Actb* loci in non-knockin newborn mice generated by injection of Cas9 protein, 0.61 pmol/μl crRNA and tracrRNA and reporter construct, and a wildtype control (WT). Sequences were aligned to a common WT sequence (common in **Figures S4, 6-7,** and **9-10** in **Additional file 1**). The 20 bp target sequences and PAM are shown. Red: Modified mice.


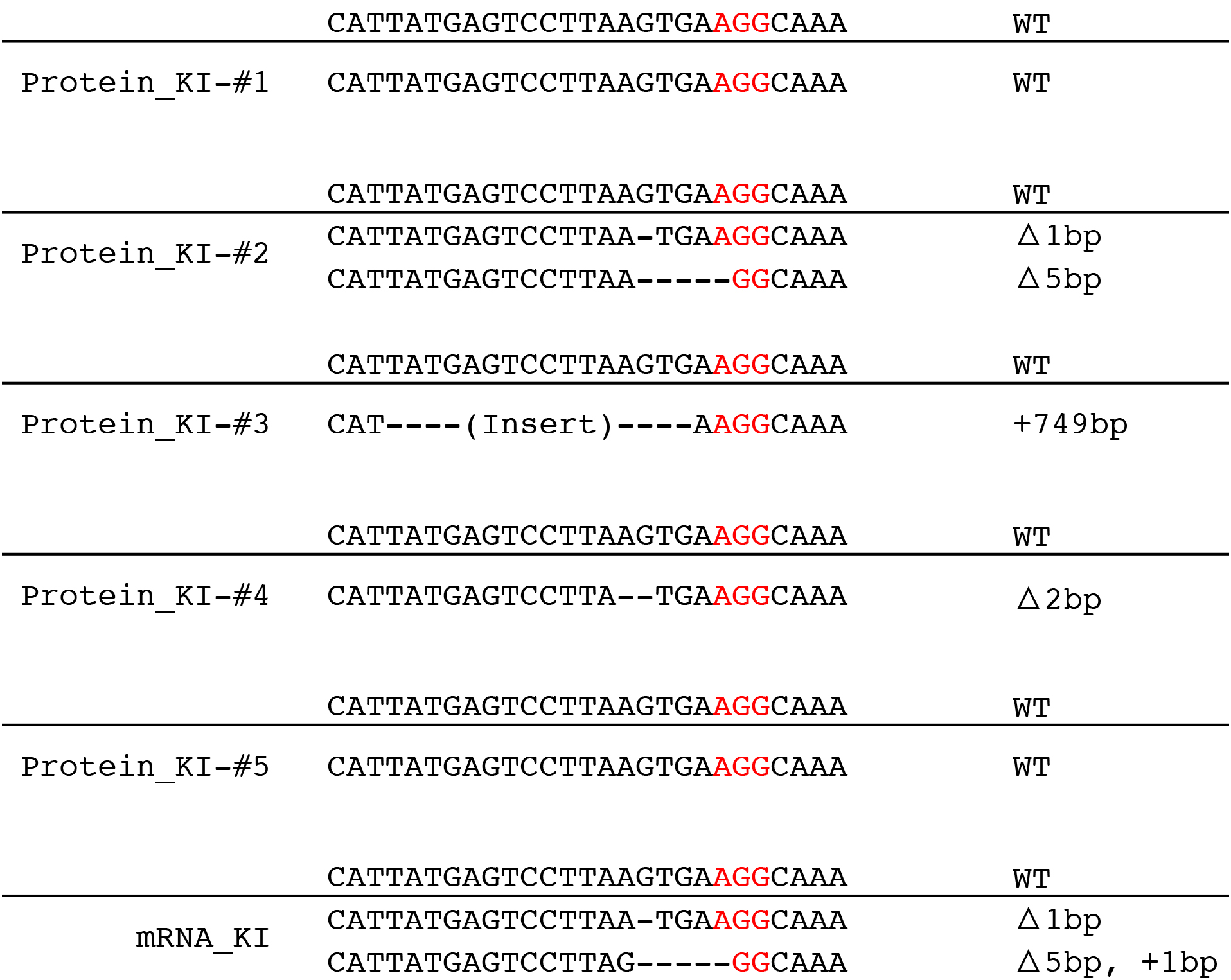


**Figure S11** Sequence analysis of non-knockin alleles of the *Actb* locus in knockin newborns. PAM sequences are labeled in red.


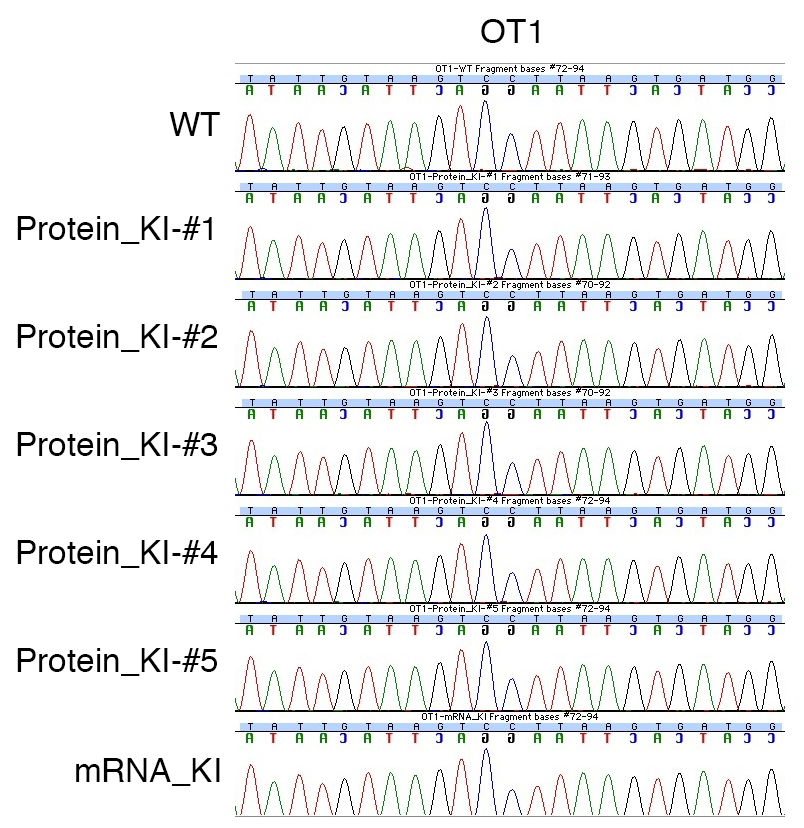


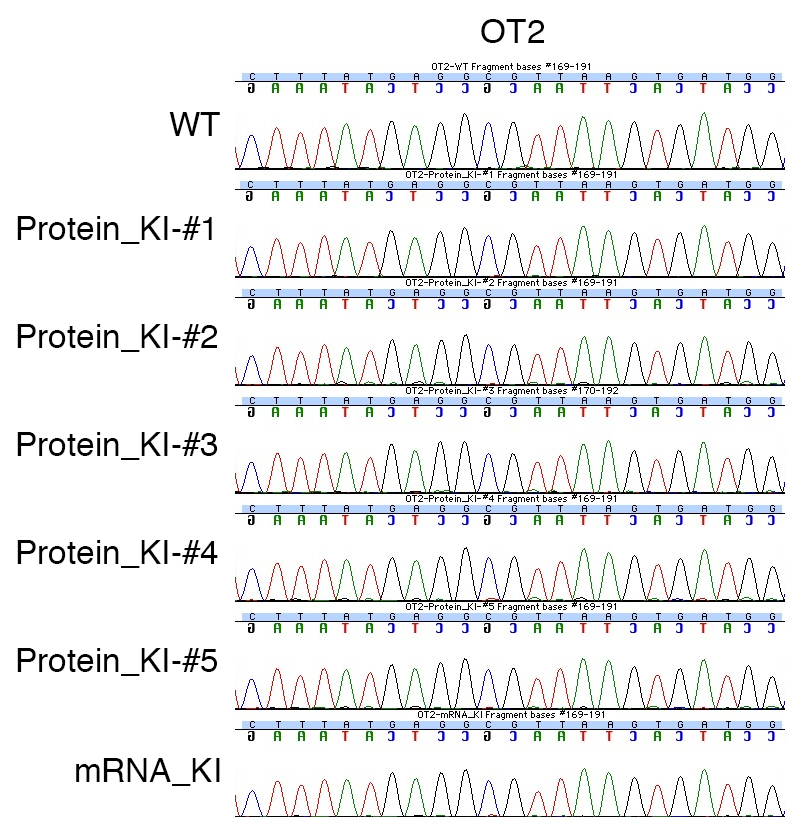


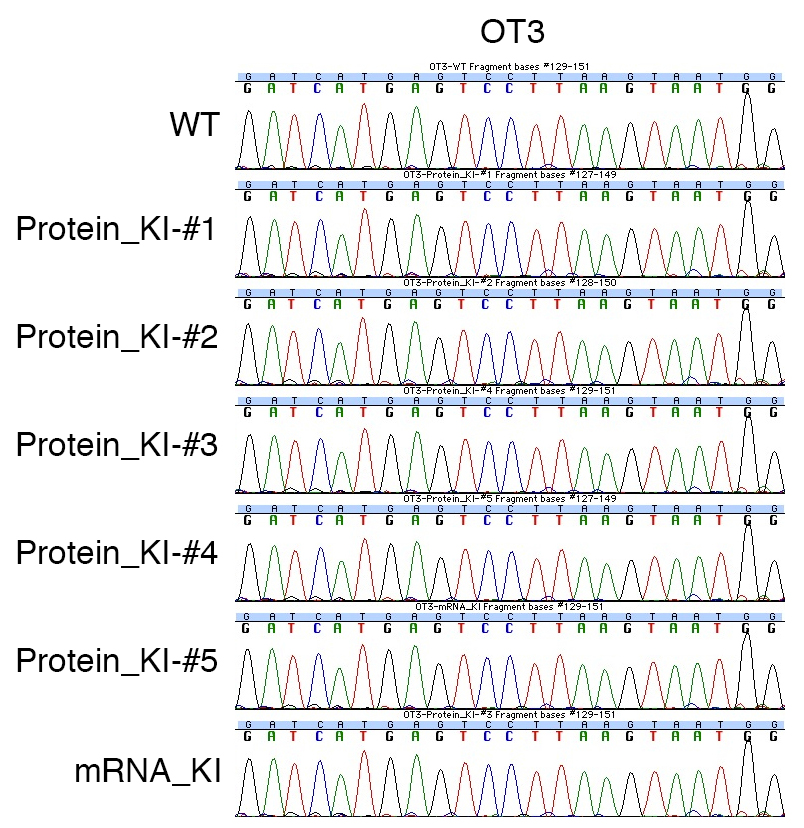


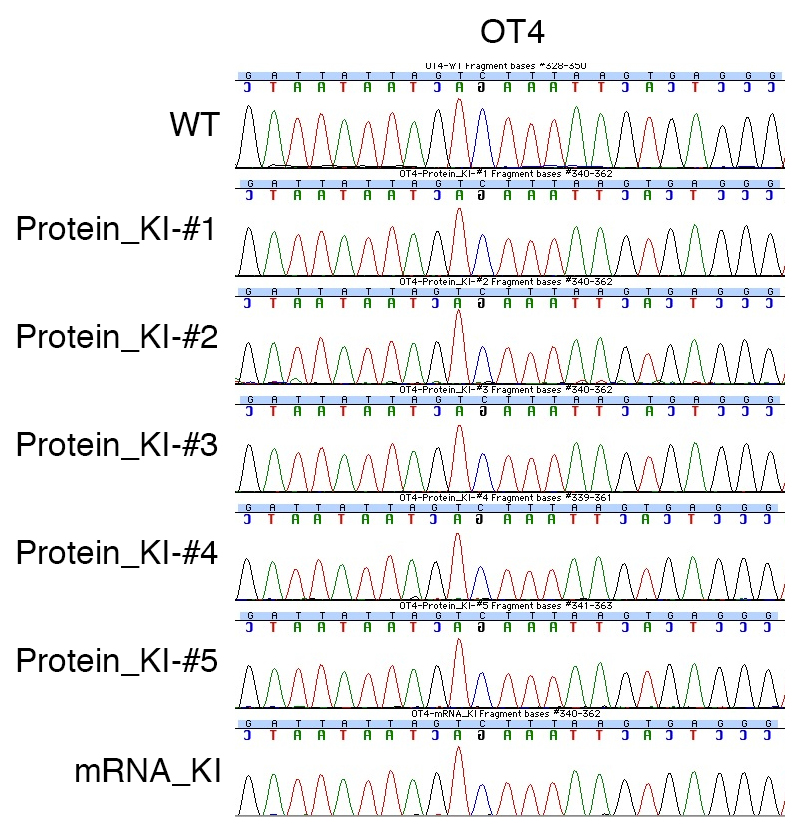


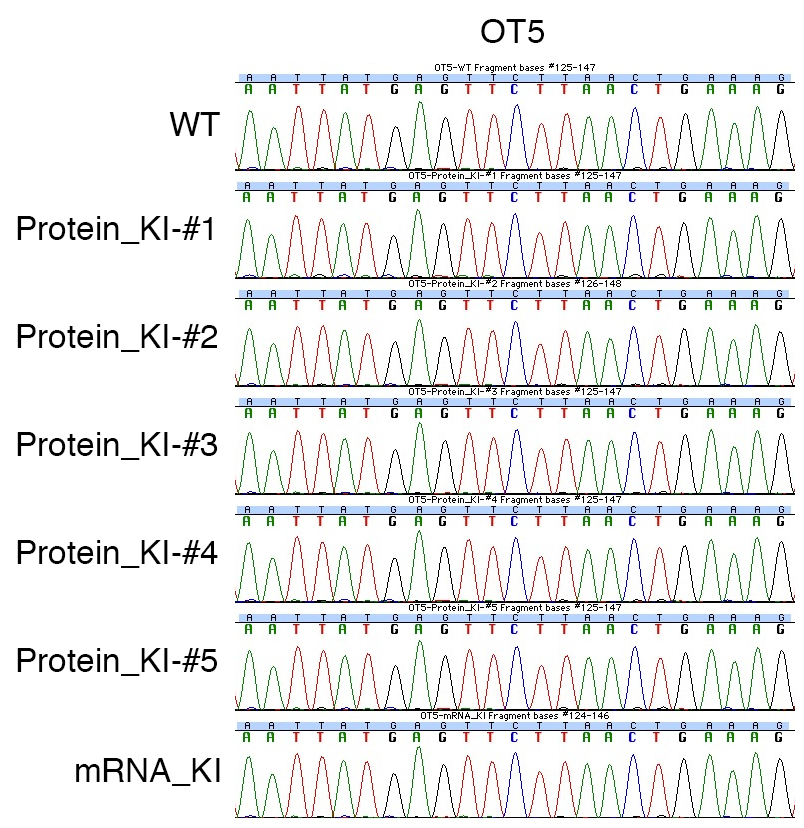


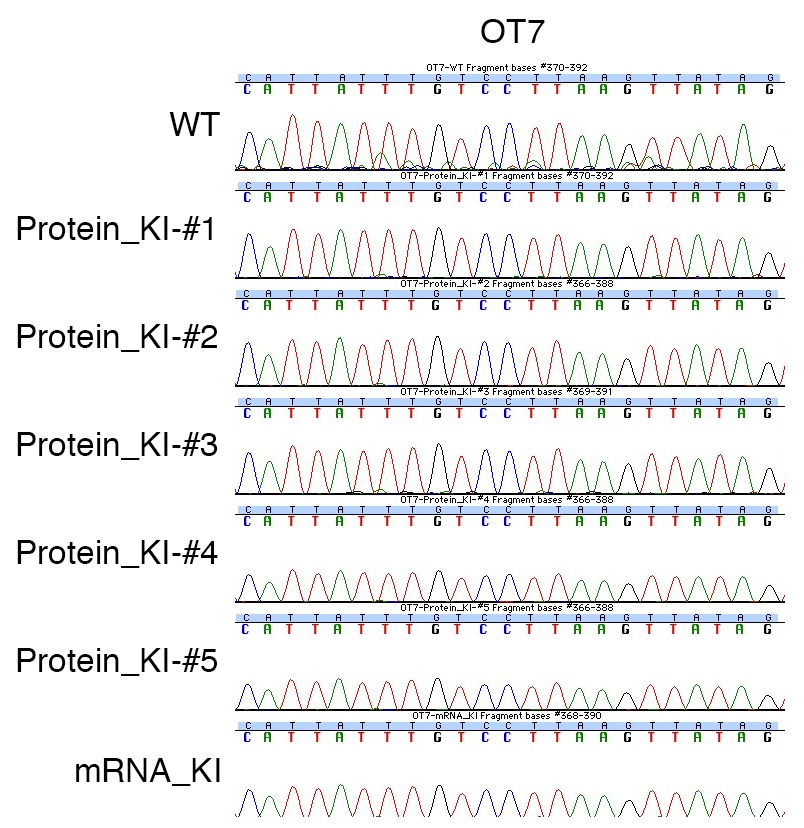


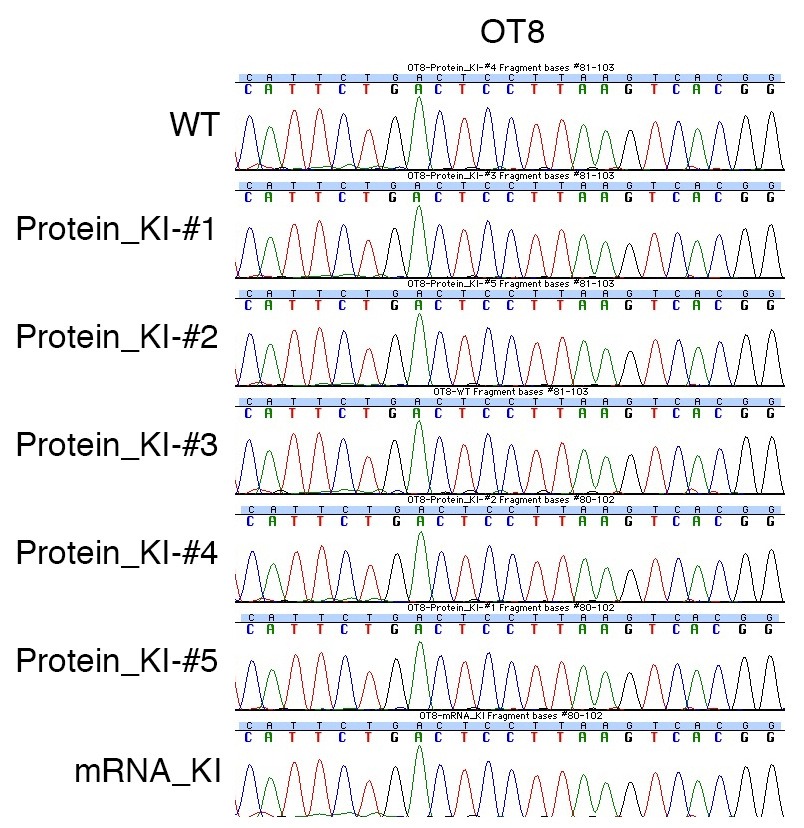


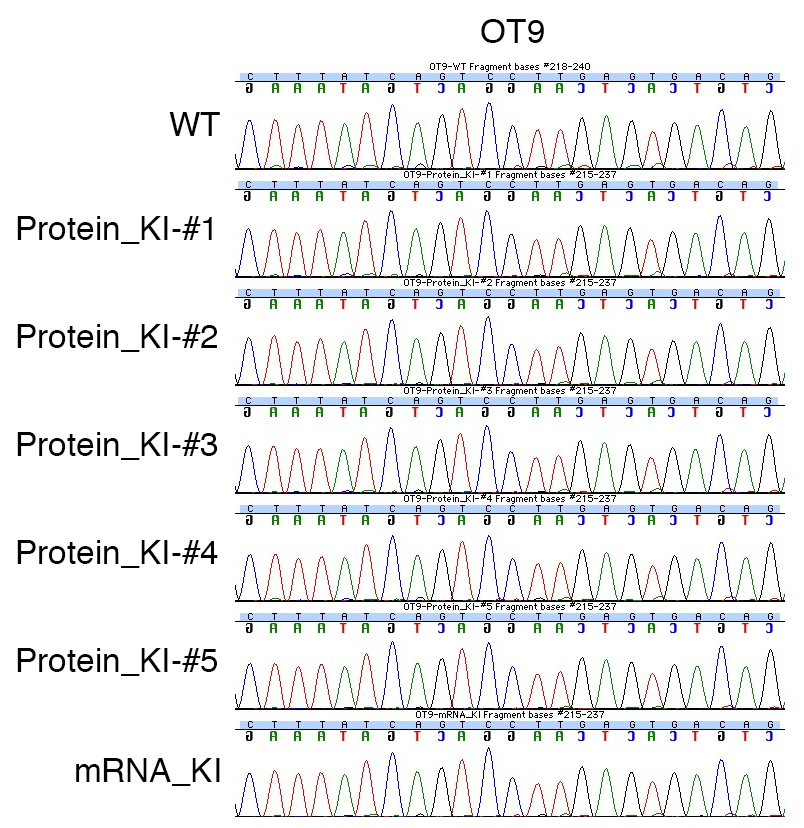


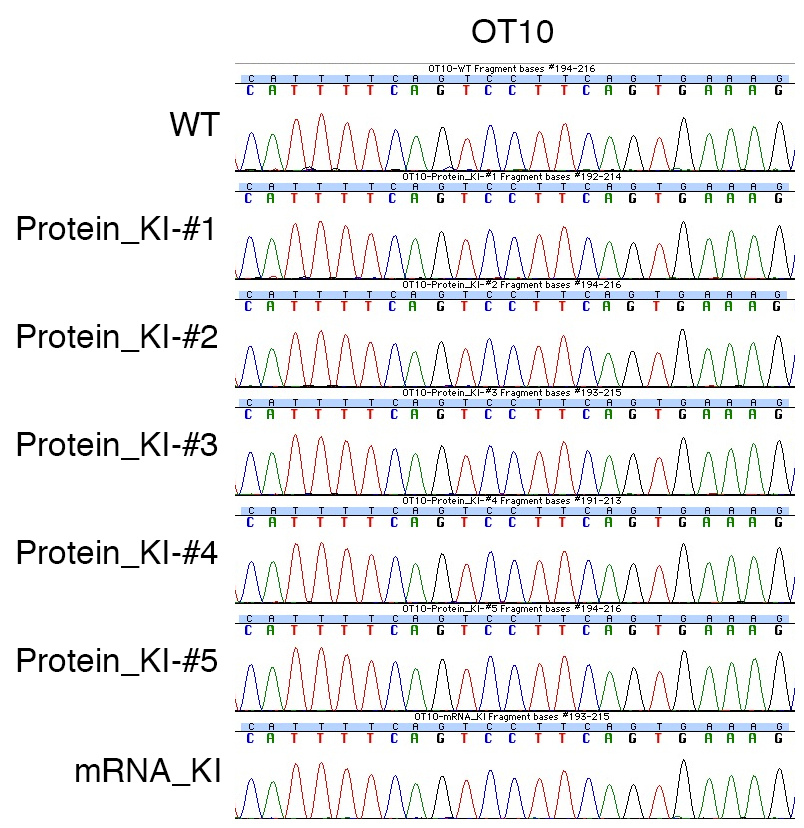


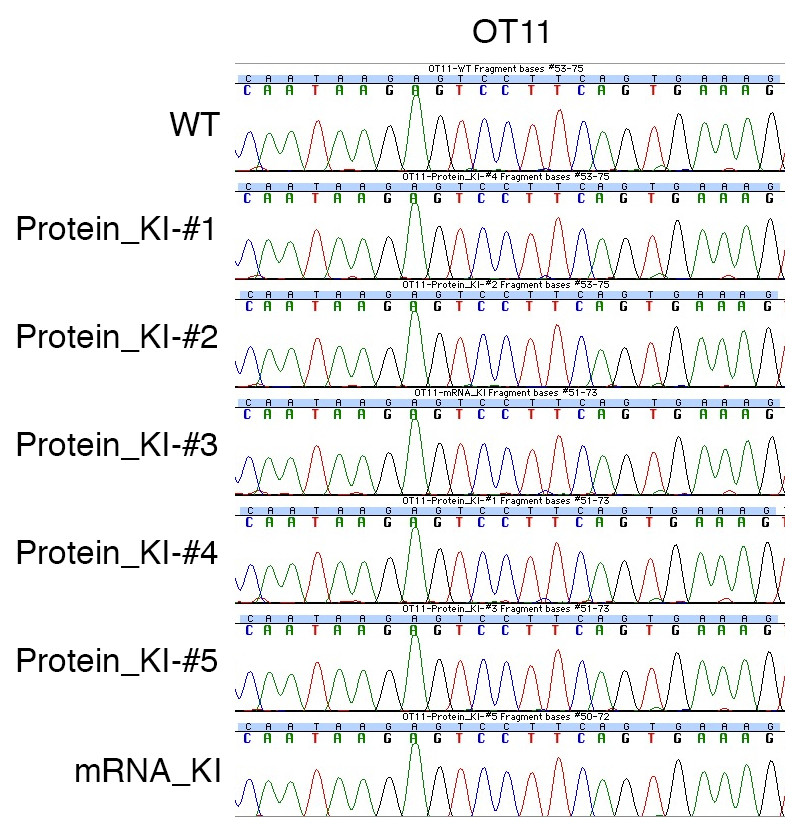


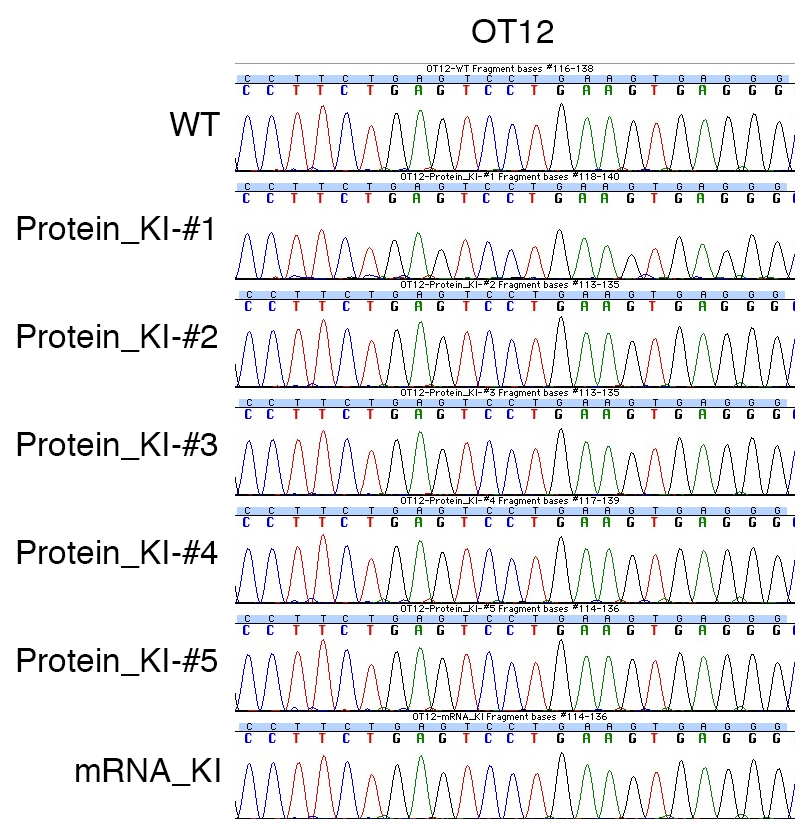


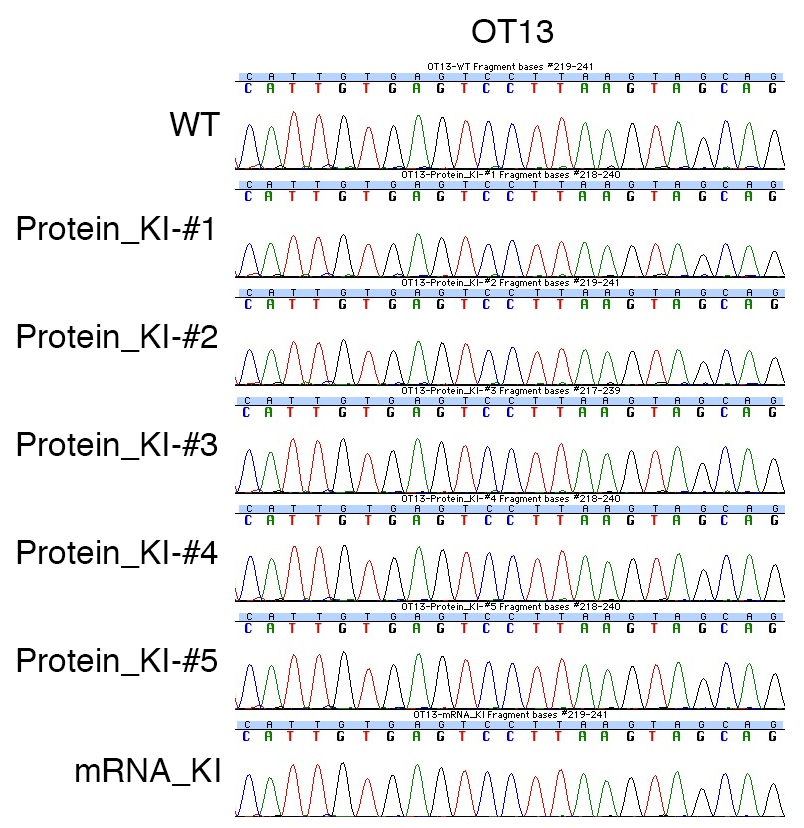


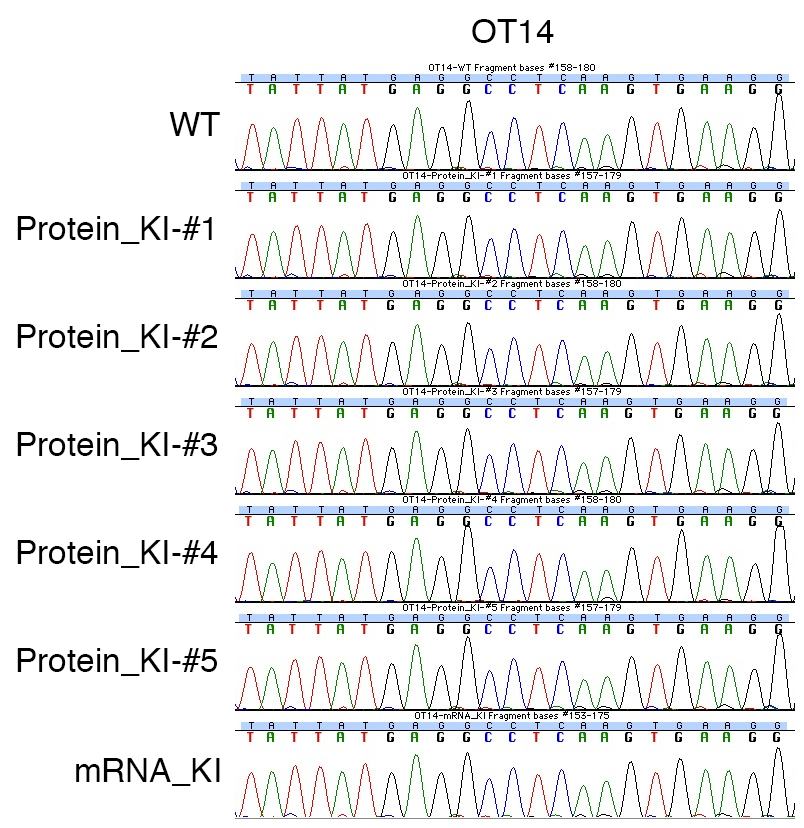


**Figure S12** Sequencing results of 13 off-target candidate loci in 6 knockin newborns and a wildtype control. The 20 bp target sequences and PAM are shown. OT6 could not be amplified by PCR.


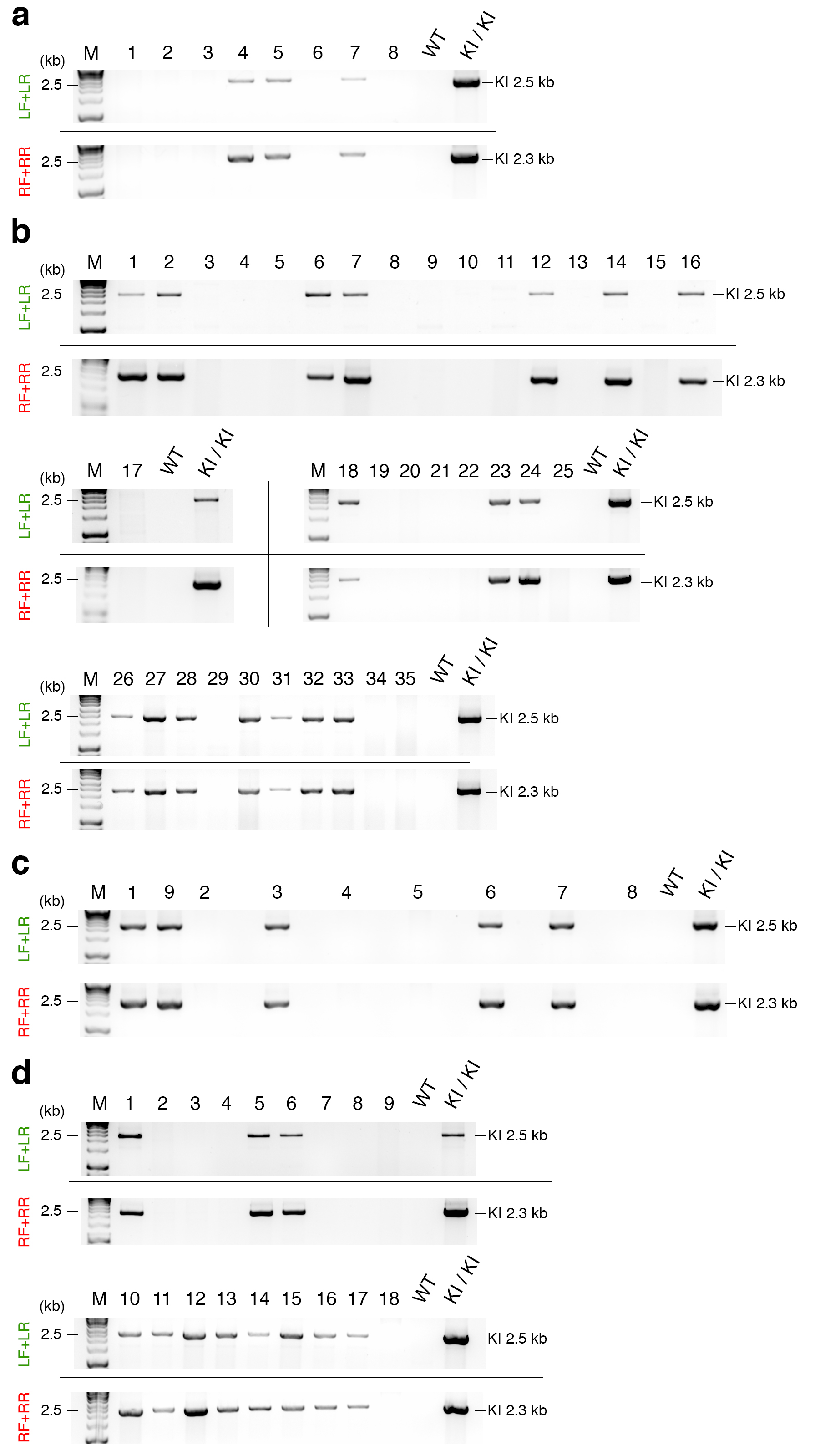


**Figure S13** Analysis of germline transmission of the knockin allele by PCR. (**a-d**) Gel images of PCR products of F1 progeny derived from (**a**) Protein_KI-#1, (**b**) Protein_KI-#3, (**c**) Protein_KI-#4, and (**d**) Protein_KI-#5 amplified with LF+LR or RF+RR primers. LF: left forward primer, LR: left reverse primer, RF: right forward primer, RR: right reverse primer, M: molecular marker, WT: wildtype, and KI: knockin.

**
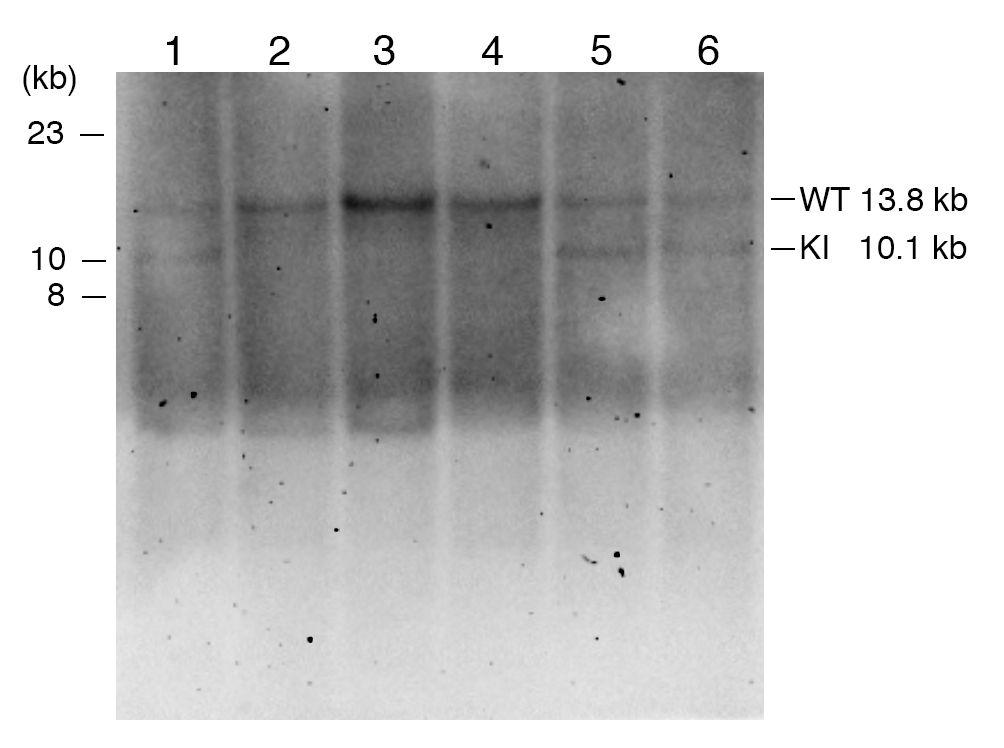
**

**Figure S14** Analysis of germline transmission of the knockin allele by southern blotting. The results of southern blotting of F1 progeny #1-6 derived from the founder Protein_KI-#5 (correspond to **Figure S13d 1-6 in Additional file 1**) are shown. WT: wildtype, and KI: knockin.

| **Injected** | **Transferred (%)** | **Newborn (%)** | **Targeted (%)** | **Bi-allelic (%)** |
| --- | --- | --- | --- | --- |
| 82 | 47 (57.3) | 12 (25.5) | 12 (100) | 11 (91.7) |

**Table S1** Generation of knockout mice by injection of Cas9 mRNA and *Actb* sgRNA. Percentages were calculated using the number in each column as the numerator and the number in the column to its left as the denominator. The percentage of Bi-allelic was calculated using the number of bi-allelically targeted newborn mice as the numerator and the number in Newborn as the denominator.

| **sgRNA (ng/μl)** | **Cas9 Protein (ng/μl)** | **Injected** | **Transferred (%)** | **Newborn (%)** | **Targeted (%)** | **Knockin (%)** |
| --- | --- | --- | --- | --- | --- | --- |
| 2.5 | 30 | 110 | 87 (79.1) | 18 (20.7) | 0 (0) | 0 (0) |
| 2.5 | 100 | 108 | 96 (88.9) | 21 (21.9) | 1 (4.8) | 0 (0) |
| 25 | 30 | 67 | 59 (88.1) | 13 (22.0) | 2 (15.4) | 0 (0) |
| 25 | 100 | 68 | 53 (77.9) | 6 (11.3) | 3 (50.0) | 0 (0) |
| Total | | 353 | 295 (83.6) | 58 (19.7) | 6 (10.3) | 0 (0) |

**Table S2** Generation of knockin mice by injection of Cas9 protein, *Actb* sgRNA, and targeting vector. Percentages were calculated using the number in each column as the numerator and the number in the column to its left as the denominator except for Knockin. Percentages of Knockin were calculated using the number in Knockin column as the numerator and the number in the Newborn column as the denominator. Note that the sum of the fourth and fifth rows is shown the third row in Table 1.

| **Dual RNA (pmol/μl)** | **Cas9 Protein (ng/μl)** | **Injected** | **Transferred (%)** | **Newborn (%)** | **Targeted (%)** | **Knockin (%)** |
| --- | --- | --- | --- | --- | --- | --- |
| 0.061 | 30 | 67 | 43 (64.2) | 9 (20.9) | 3 (37.5)* | 0 (0) |
| 0.61 | 30 | 107 | 65 (60.7) | 11 (16.9) | 9 (81.8) | 5 (45.5) |
| Total | | 174 | 108 (62.1) | 20 (18.5) | 12 (63.2)* | 5 (25.0) |

**Table S3** Generation of knockin mice by injection of Cas9 protein, *Actb* crRNA, tracrRNA, and targeting vector. Percentages were calculated using the number in each column as the numerator and the number in the column to its left as the denominator except for Knockin. Percentages of Knockin were calculated using the number in Knockin column as the numerator and the number in the Newborn column as the denominator. *The PCR product of #4 mouse could not be sequenced, thus the percentages of Targeted were calculated using 8 or 19 as the denominator. Note that the result of the third row is same to that of Protein + Dual RNA in Table 1.


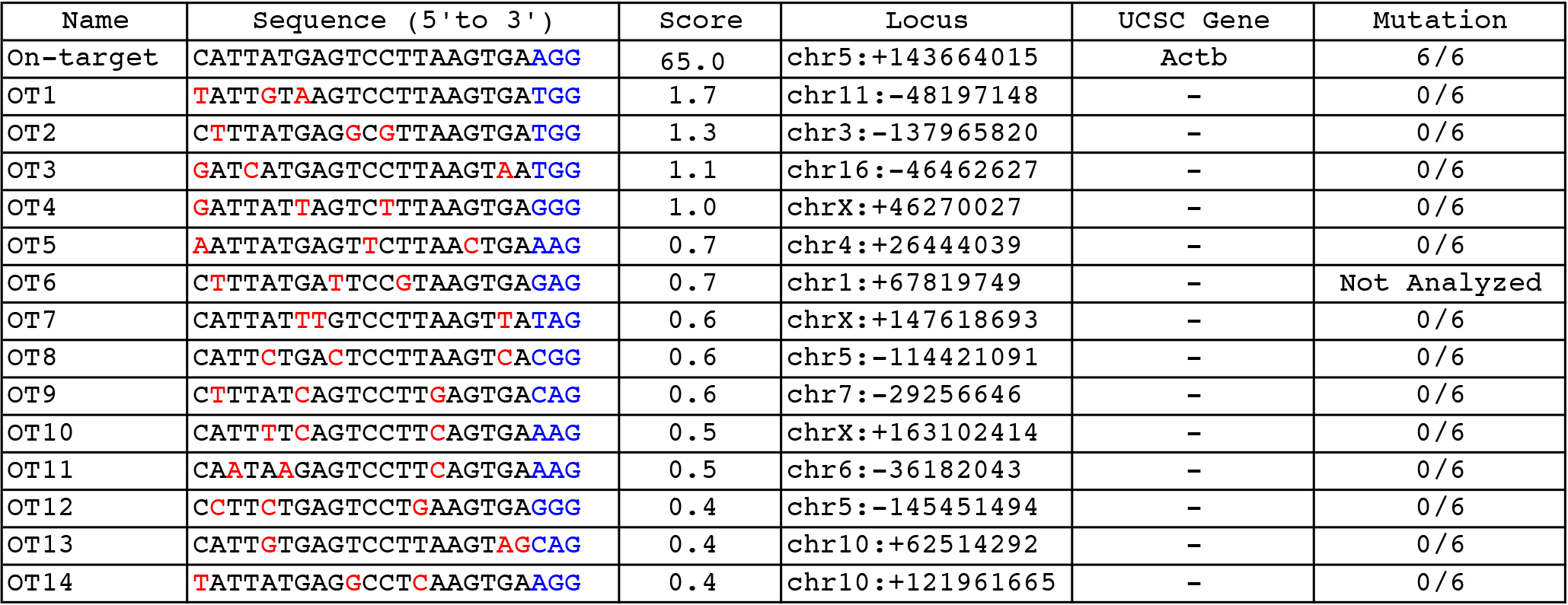


**Table S4** Mutations at putative off-target loci containing up to 3 bp mismatches for *Actb* target are analyzed by sequencing. Mismatches compared to on-target sequence are shown in red. PAM sequences are labeled in blue.

| **Knockin Line** | **Sex** | **Mating Strain** | **F1 Newborn** | **F1 Knockin (%)** |
| --- | --- | --- | --- | --- |
| Protein_KI-#1 | Female | BDF1 | 8 | 3 (37.5) |
| Protein_KI-#3 | Male | C57BL/6 | 35 | 17 (48.6) |
| Protein_KI-#4 | Female | BDF1 | 9 | 5 (55.6) |
| Protein_KI-#5 | Female | C57BL/6 | 18 | 11 (61.1) |
| Total | - | - | 70 | 36 (51.4) |

**Table S5** The number of F1 progeny carrying the transgene for four founder knockin mice. Percentages were calculated using the number in each column as the numerator and the number in the column to its left as the denominator.


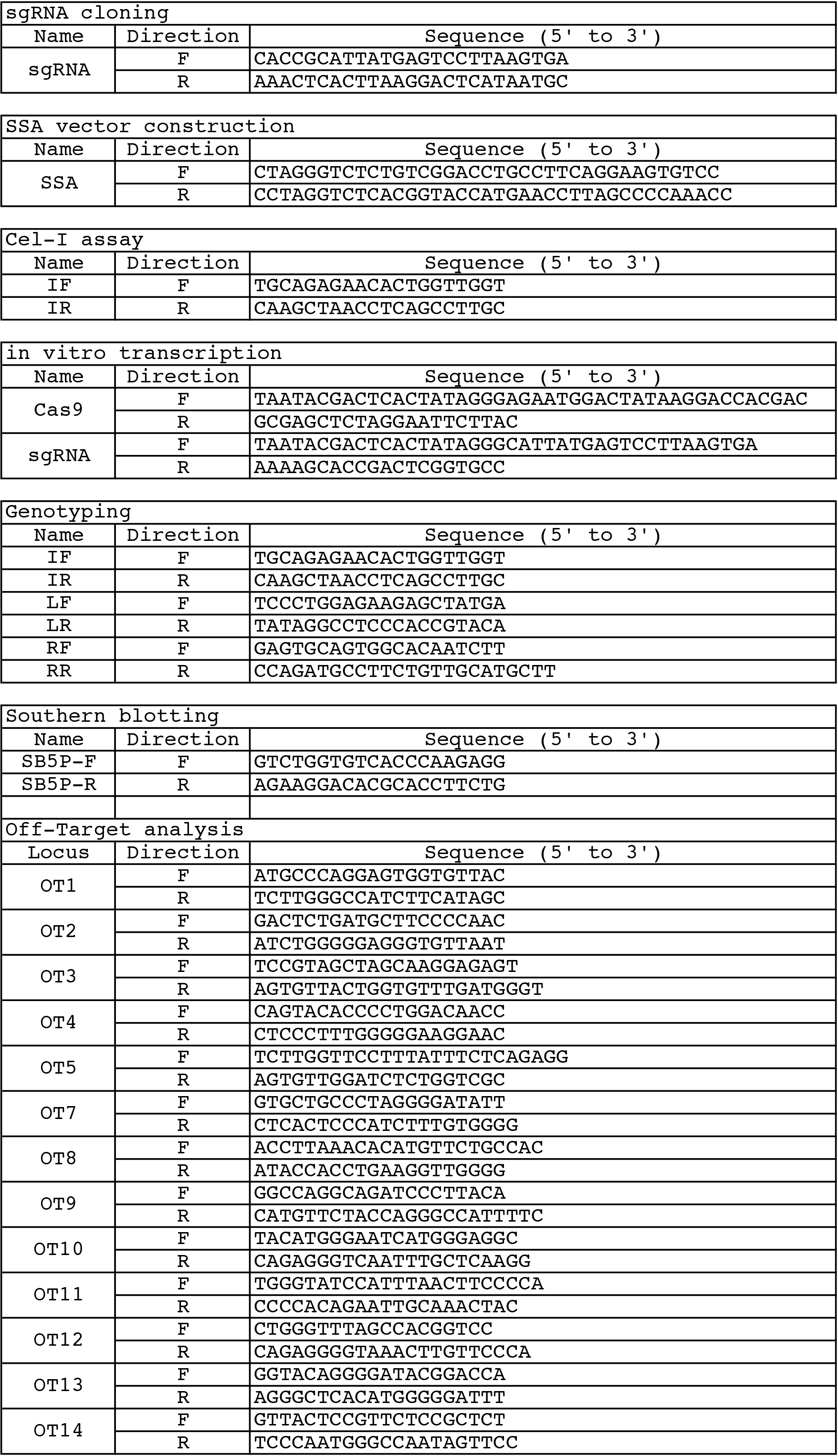


**Table S6** Oligo DNAs and primers used in this study.
